# Subcellular spatial regulation of immunity-induced phosphorylation of RIN4 links PAMP-triggered immunity to Exo70B1

**DOI:** 10.3389/fpls.2024.1473944

**Published:** 2024-12-13

**Authors:** Yi Zhao, Brad Day

**Affiliations:** ^1^ Department of Plant, Soil and Microbial Sciences, Michigan State University, East Lansing, MI, United States; ^2^ Plant Resilience Institute, Michigan State University, East Lansing, MI, United States; ^3^ Graduate Program in Genetics and Genome Sciences, Michigan State University, East Lansing, MI, United States; ^4^ Graduate Program in Molecular Plant Sciences, Michigan State University, East Lansing, MI, United States

**Keywords:** RIN4, plasma membrane localization, EXO70B1, phosphorylation at T166, PTI

## Abstract

RIN4 is a crucial regulator of plant immunity, playing a role in both PAMP-triggered immunity (PTI) and effector-triggered immunity (ETI). While the impact of post-translational modifications (PTMs) on RIN4 has been extensively studied, their specific effects on plant immune response regulation and the underlying mechanisms have remained unclear. In this study, we investigated the phosphorylation of RIN4 at threonine-166 (RIN4^T166^) in *Arabidopsis* transgenic lines expressing various RIN4 variants. Our pathological and molecular genetic analyses reveal that RIN4^T166^ phosphorylation disrupts its localization to the plasma membrane (PM) and represses plant defense activation. We found that RIN4’s PM tethering relies on Exo70B1-mediated exocytosis and the integrity of the host cytoskeletal actin network. Phosphorylation at RIN4^T166^ disrupts its PM localization due to reduced binding affinity with Exo70B1. This disruption was further evidenced by the *35S::RIN4^T166D^/rin124* transgenic line, which exhibited suppressed PTI responses similar to the *exo70b1* mutant. Our findings demonstrate that RIN4’s subcellular localization is regulated by phosphorylation, suggesting that plants use a sophisticated network of signaling processes to precisely control the timing and localization of immune signaling activation. This study uncovers a mechanism by which PTI is repressed through RIN4 phosphorylation, providing new insights into the spatial regulation of RIN4 within plant immune signaling pathways.

## 1 Introduction

Despite lacking specialized immune cells, plants possess a highly effective defense system that utilizes a suite of physical and chemical mechanisms to protect themselves against pathogen attack (Li et al., 2020). Once pathogens pass host physical barriers, such as waxy cuticle and the cell wall, they encounter the first line of the genetically-encoded plant innate system, which includes a constellation of PM-anchored pattern recognition receptors (PRRs) whose function is to detect microbe-associated and/or damage-associated molecular patterns (MAMPs and DAMPs) (McMillan et al., 2021; Ngou et al., 2021b). This first layer of the plant immune system, referred to as pattern-triggered immunity (PTI), relies on the activity of PRRs, which includes receptor kinase (RLKs) and receptor-like proteins (RLPs) to initiate a rapid, robust, cellular defense system (Zipfel, 2014). Once MAMPs/DAMPs are sensed by the extracellular domain of PRRs, the intracellular domain of the PRR initiates signal transduction and triggers PTI through downstream signaling processes amplified by protein-protein interactions (Monaghan and Zipfel, 2012).

In parallel to the activation of PTI, and in response to the activation of host defenses, pathogens deploy a constellation of virulence effector proteins to interfere with PTI (Jones and Dangl, 2006). The outcome of this is the initiation of effector-triggered susceptibility (ETS), which results in the downregulation of immune signaling and additional key physiological processes required for plant growth and survival (Nomura et al., 2006). To counter the virulence activity of these secreted effectors, plants evolved a second layer of innate immunity, termed effector-triggered immunity (ETI). In brief, ETI is driven by intracellular immune receptors, the majority of which are nucleotide-binding site leucine rich repeat (NLR) proteins, which are activated after perceiving secreted virulence effectors directly or indirectly (Baggs et al., 2017). Despite PTI and ETI having significant differences in their modes of activation (Tsuda et al., 2009; Gu et al., 2016), the two innate immune responses share numerous points of convergence, and thus, function in a mutualistic manner to potentiate host defenses in response to pathogen infection (Thomma et al., 2011; Ngou et al., 2021a; Wang et al., 2023; Yu et al., 2024).

RPM1-interacting protein 4 (RIN4) is a small unstructured protein tethered to the PM (Takemoto and Jones, 2005), where it functions in signaling processes associated with both PTI and ETI (Zhao et al., 2021). RIN4 negatively regulates PTI since plants lacking RIN4 show enhanced PTI responses (Kim et al., 2005; Liu et al., 2009). RIN4 also negatively regulates ETI by interacting with intracellular immune receptors that associate with the activation of ETI (Kim et al., 2005). Phosphorylation of RIN4 is a key post-translational mechanism that regulates its cellular function (Toruno et al., 2019). Indeed, the phosphorylation status of RIN4 is guarded by the cell’s immune system and underpins its multiple interactions and functions during pathogen infection (Ray et al., 2019). AvrB and AvrRpm1, two specific pathogen type-III effector proteins (T3Es), induce the accumulation of the PM-associated receptor-like cytoplasmic kinases (RLCKs) which subsequently activate the NLR receptor RPM1 (Chung et al., 2011; Xu et al., 2017; Yang et al., 2021). It has been reported that RPM1-induced protein kinase (RIPK) phosphorylates RIN4 at three sites: Thr21, Ser160, and Thr166. Phosphorylation at T166 initiates RPM1-activated HR, whilst dephosphorylation suppresses the activation of RPM1. Together, these data support the hypothesis that the RIN4^T166D/E^ phospho-switch is required for the activation of ETI (Liu et al., 2011). Interestingly, the flagellin peptide flg22 triggers phosphorylation of RIN4 at Ser-141 (pS141), which results in an enhanced PTI response. Although the function of RIN4 pS141 could be counteracted by the pT166 of RIN4, the mechanism still remains elusive (Chung et al., 2014).

Membrane trafficking contributes to the establishment of rapid and highly specific immune responses, in large part through regulation of the subcellular localization of plant immunity-related proteins (Liu et al., 2021; Xing et al., 2021). The exocyst is a multi-protein complex comprised of eight subunits (Sec3, Sec5, Sec6, Sec8, Sec10, Sec15, Exo84, and Exo70), and as a critical component of plant immune system function, is required for defense cargo secretory vesicles docking to the PM (Mei and Guo, 2018). In *Arabidopsis*, 23 members of the EXO70 family have been identified, illustrating the breadth and diversity of functions in plant immune signaling (Pecenkova et al., 2020; Wang et al., 2020; Brillada et al., 2021). Among these members, Exo70B1 has been demonstrated to be necessary for the atypical NLR protein TN2 (Toll/interleukin-1 receptor–nucleotide-binding sequence protein2)-mediated ETI response, and is hypothesized to mediate immune signaling activation via a guard hypothesis-based mechanism (Hou et al., 2020). Indeed, Exo70B1 has been shown to be targeted by the *Pseudomonas* effector AvrPtoB, resulting in Exo70B1 ubiquitination and consequent degradation (Wang et al., 2019). In the knockout mutant *exo70B1-3*, NLR-mediated ETI is constitutively activated (Zhao et al., 2015). Interestingly, Exo70B1 has also been reported to regulate PTI responses by maintaining the proper abundance of FLS2 at the PM (Ruano and Scheuring, 2020).

Previous studies have established a mechanistic link between Exo70B1 and RIN4, demonstrating that *Arabidopsis* Exo70B1 physically interacts and co-localizes with *Arabidopsis* RIN4 at the PM in *Nicotiana benthamiana* (Sabol et al., 2017). However, the *in vivo* biological significance of this interaction remains uncertain. In this study, we examined whether the PM localization of RIN4 is essential for its role in PAMP-triggered immunity (PTI) and effector-triggered immunity (ETI). We also investigated whether the interaction between Exo70B1 and RIN4 is crucial for RIN4-mediated plant immunity. Our findings reveal that disrupting RIN4’s membrane localization represses PTI. Additionally, phosphorylation at RIN4^T166^ alters RIN4’s PM localization by preventing its interaction with Exo70B1. The deletion of *Exo70B1*, similarly, disrupts RIN4’s proper localization and compromises PTI. Taken together, these results offer a comprehensive view of the dynamic regulation of RIN4 during host-microbe interactions, providing deeper insights into the spatiotemporal organization of innate immune responses.

## 2 Materials and methods

### 2.1 Plant material and growth conditions

*Arabidopsis thaliana* Col-0 ecotype (i.e., WT Col-0) was used in all experiments. Mutants and transgenic lines used in this study include *rpm1/rps2/rin4* (*r124*), *rpm1/rps2/rin4*/*exo70B1-1* (*r124b1*), *rpm1-3/rps2-101c/exo70B1-1* (*r12b1*), *gRIN4p::T7-RIN4/r124*, *gRIN4p::*T7-RIN4^T166A^ */r124*, *gRIN4p::T7-RIN4^T166D^/r124*, *r124* co-expressing *35S::*RFP-PIP2A and YFP fused RIN4 variants (RIN4, RIN4^T166A^, and RIN4^T166D^), *r124* co-expressing *35S::*RFP-ST and YFP fused RIN4 variants (RIN4, RIN4^T166A^, and RIN4^T166D^), *35S::YFP-RIN4/r124b1*, *35S::*YFP-RIN4^T166A^ */r124b1*, *35S::YFP-RIN4^T166D^/r124b1*, *exo70B1-1* (CS2103358, ABRC), *exo70B1-2* (CS414954, ABRC), *exo70B2* (CS2103357, ABRC), *exo70B1-1*/*exo70B2* (CS2103356, ABRC), *35S::YFP-RIN4*/Col-0, *35S::YFP-RIN4/r124*, *35S::YFP-RIN4^T166A^/r124*, *35S::YFP-RIN4^T166D^/r124*, *35S::YFP-RIN4*/*exo70B1-1*, and *35S::HA-RIN4^T21E/S160E/T166D^/r124*. To generate transgenic lines expressing YFP- or HA-tagged RIN4 derivatives, the coding sequence of *RIN4* was inserted into pENTR/D-TOPO followed by site-directed mutagenesis using the Phusion Site-Directed Mutagenesis Kit (Thermo Scientific). The mutated and non-mutated coding sequences of RIN4 were subsequently recombined into the pEarleyGate104 or pGWB515 binary vector with the Gateway LR Clonase Kit (Thermo Scientific). The final constructs were introduced into WT Col-0, or mutants plants (e.g., *r124*, *exo70B1-1*), using the floral dip method (Zhang et al., 2006). The seeds of T7-tagged lines were provided by Dr. Jeff Dangl (UNC-Chapel Hill, USA). Mutants were identified by PCR and DNA sequencing. Primers used for genotyping are listed in **Supplementary Table S1**. *Arabidopsis* and *Nicotiana benthamiana* plants were grown in a growth room at 20°C and ~60% relative humidity in a 12-h-light/12-h-dark day/night cycle.

### 2.2 Transient protein expression in protoplasts and confocal microscopy assay

For transient protein expression analyses, leaf mesophyll protoplasts were isolated from 4-week-old *Arabidopsis* plants and transfected using the polyethylene glycol method (Wu et al., 2009). Microscopic images were captured under a fluorescence microscope (Nikon A1R-TIRF-STORM) that equipped with a 40x oil objective after 16 h. For CHX (cycloheximide) treatments, methods previously described were followed (Maldonado-Bonilla et al., 2014). For the Latrunculin-B (LatB) treatment assay, protoplasts were treated with 0.1 μM LatB for 30 minutes at room temperature (ca. 25°C). For the stable transgenic lines expressing fluorescence protein, the cotyledons were visualized under a Nikon A1R-TIRF-STORM fluorescence microscope. The fluorescence of YFP, GFP, RFP, and chlorophyll were observed using the following excitation/emission wavelengths: 513/520-550 nm, 488/500-525 nm, 561/580-620 nm, and 488/680-700 nm, respectively. The fluorescence intensity was measured by using the “Measure” tool, Fiji software (https://fiji.sc/), and data were analyzed using GraphPad Prism 6 software.

### 2.3 *In planta* bacterial growth assay

A solution of *Pst* DC3000 *hrcC^-^* bacteria were syringe-infiltrated into leaves of 4-week-old *Arabidopsis* plants (final OD_600nm_ = 0.002). Bacterial growth was determined at 0- and 3-days post-inoculation. The flg22-activated bacterial growth suppression assay was performed as described by Knepper et al (Knepper et al., 2011). Four-to-five-week-old *Arabidopsis* plants were hand-infiltrated with 1 µM of flg22 or distilled water on the lower leaf surface, *Pst* DC3000 bacteria were hand-infiltrated at 1×10^6^ cfu/mL (OD_600nm_ = 0.002) 24 h post flg22-treatment. Bacterial growth was determined 2-days post-inoculation. Statistical difference in bacterial growth was analyzed by pairwise comparisons for all means using one-way ANOVA with GraphPad Prism 6 software.

### 2.4 RNA isolation and real-time PCR analysis

Total RNA was extracted using the RNeasy Plant Mini Kit (Qiagen) and converted to first-strand cDNA using the SuperScript III reverse transcriptase Kit (Invitrogen). The *IPP2* gene (At3g02780) was used as the normalization standard (Marcolino-Gomes et al., 2015) and the comparative Ct method (2^-ΔΔ^ *^C^* ^t^). The primers used for RT-PCR are listed in **Supplementary Table S1**. The expression levels of *RIN4, Exo70B1, and Exo70B2* were analyzed with GraphPad Prism 6 software.

### 2.5 Protein extraction and western blot analysis

Plant tissue samples were prepared as previously described. Total PM protein was extracted following the kit protocol (Minute Plasma Membrane Protein Isolation Kit for Plants, Invent Biotechnologies). Golgi were isolated with Minute™ Plant Golgi Apparatus Enrichment Kit (Invent Biotechnologies). Protein samples were resolved by electrophoresis on 4-12% Bis-Tris Protein gels. After transferring to nitrocellulose membranes, the membranes were blocked in 5% nonfat dry milk diluted in phosphate-buffered saline (PBS) for 1-2 h at room temperature. The membrane was then transferred into a 5% dry milk solution containing primary antibody and incubated for 1 h at room temperature. After washing with PBS buffer for 10 minutes, 3 times, the membrane was incubated in incubation solution with secondary antibody (1:10,000) for 1 h and then washed with PBS containing 1% Tween20 for 10 minutes, 3 times. The SuperSignal West Pico PLUS Chemiluminescent Substrate Kit (Pierce) was used to detect the protein signal. Actin was detected using an anti-Actin antibody (Sigma-Aldrich; cat. #A0480, 1:2000). RIN4 was detected using the anti-RIN4 antibody (1:2000). YFP was detected using the anti-GFP antibody (Sigma, SAB4301138; 1:2000). Golgi were detected using the anti-Sec21p antibody (Agrisera, AS08 327; 1:2000).

### 2.6 Yeast two-hybrid analysis

The full-length RIN4 coding sequence, as well as those of the phospho-derivatives of RIN4 (e.g., RIN4^T166D^, and RIN4^T21ES160ET166D^), were cloned into the bait vector *pDEST32*. *EXO70B1* was cloned into the prey vector *pDEST22*. The interaction between RIN4 and *pDEST22* empty vector, and the interaction between *pDEST32* empty vector with *Exo70B1*, served as negative controls. The interaction between RIN4 and AHA1 was used as positive control. The bait and prey vectors were co-transformed into the yeast strain AH109 in different combinations, and the transformants were selected on synthetic dextrose (SD) medium without Leu and Trp (SD-Leu-Trp). Positive clones were plated on SD-Leu-Trp-Ura-His medium.

### 2.7 Bimolecular fluorescence complementation assay

*Agrobacterium tumefaciens* strain GV3101 expressing the desired constructs (*pSPYNE::*RIN4, *pSPYCE::*Exo70B1, and *pSPYCE::*Exo70A1) were diluted in infiltration buffer (10 mM MgSO_4_·7H_2_O with 100 µM acetosyringone) at an optical density OD_600nm_ = 0.05. The *Agrobacterium* mixture was infiltrated into the abaxial leaf surface of 2-week-old *N. benthamiana* leaves, as previously described (Sassmann et al., 2018). Fluorescence was observed at 72 h post-infiltration as previously described.

### 2.8 MAPK assay

Plants were grown for 4 weeks, and fully expanded leaves were infiltrated with flg22 (1 µM). Leaf samples were then collected at 0, 5-, 10-, 15-, and 30-min time points for WT Col-0, *exo70B1-1*, *exo70B1-2*, *exo70B2*, and *exo70B1-1*/*exo70B2*. Each sample weighed 0.1 g and then was ground in liquid nitrogen. After grinding, 100 µl protein extraction buffer (50 mM Tris–HCl (pH 7.5), 5 mM EDTA, 5 mM EGTA, 150 mM NaCl, 1 mM DTT, 10 mM Na_3_VO_4_, 10 mM NaF, 50 mM β-glycerolphosphate, 1 mM phenylmethylsulfonyl fluoride (PMSF), plant protease inhibitor cocktail (Sigma-Aldrich), 10% glycerol, 0.1% NP-40) for each tube was added and well mixed immediately. After centrifugation, the protein supernatant was mixed with Laemmli sample buffer and boiled for 5 min.

MAPK activity was detected by western blotting using p44/42 MAPK (Erk1/2) Antibody (Cell Signaling Technology, #9102S; dilution, 1:1000) as primary antibody and goat anti-rabbit-HRP (Amersham Biosciences) as secondary antibody. Actin was detected using an anti-actin antibody (Sigma-Aldrich; cat. #A0480) as the primary antibody and goat anti-mouse IgG (H+L) as the secondary antibody (Invitrogen; cat. #31430).

### 2.9 ROS assay

The leaf discs from the fully expanded leaves of 4-week-old WT Col-0, *fls2*, *exo70B1-1*, *exo70B1-2*, and *exo70B1-1*/*exo70B2* were placed into a white 96-well plate, which was with 150 μl of water per well. After overnight incubation, 100 μl of reaction mixture (17 μg/mL of Luminol (Sigma), 10 μg/mL of Horseradish Peroxidase (HRP; Sigma), 100 nM flg22), was substituted for the water to monitor ROS burst (Chung et al., 2014). Luminescence was measured immediately with 1 s integration and 2 min interval over 60 min by using a BioTek Synergy H1 plate reader. Every leaf was sampled for only one disc of 20 mm diameter, each plant was punched for two leaf discs, and eight plants of each line were used for sampling. GraphPad Prism 6 was used for data analysis.

### 2.10 Immunogold labelling assay and transmission electron microscope analysis

To investigate the subcellular localization of RIN4 and the RIN4 phospho-variants *in planta*, the fully expanded leaves of 4-week-old plant were dissected into 1 mm^2^ leaf discs in fixation solution (4% (v/v) paraformaldehyde and 0.5% (v/v) glutaraldehyde in PBS buffer), followed by the application of a vacuum ensure complete infiltration of the fixative. The samples were embedded in LR White (Zechmann et al., 2005). Ultra-thin sections (80 nm) were collected on formvar-coated nickel grids. The grids were pre-incubated with the blocking solution (0.01M PBS, 5% BSA, and 5% normal goat serum) for 10-30 minutes at room temperature, then washed three times with incubation buffer (0.01M PBS and 0.1% BSA) for 5 minutes each. The grids were then incubated with the primary antibody (anti-RIN4 antibody, diluted 1:500 in incubation buffer) for 1 hour at room temperature. Afterward, the grids were washed three times with incubation buffer (5 minutes per wash), followed by incubation with the secondary antibody (Goat anti-mouse IgG coupled with 10 nm colloidal gold; Sigma, G7652), which was diluted 1:20 in incubation buffer, for 1 h. The grids were washed in incubation buffer three times (5 minutes each), followed by three washes in distilled water (5 minutes each). Ultra-thin sections were stained with uranyl acetate followed by lead citrate. The samples were embedded and cut into sections in the Center for Advanced Microscopy (CAM) at Michigan State University. Immunogold labeling and TEM analyses were also conducted in the CAM. The measurements of the size of RIN4 aggregation particles were performed using Fiji (https://fiji.sc/). The data were analyzed using GraphPad Prism 6.

## 3 Results

### 3.1 The phosphorylation status of RIN4 modulates its PM-localization and the suppression of PTI

**Figure 1:**
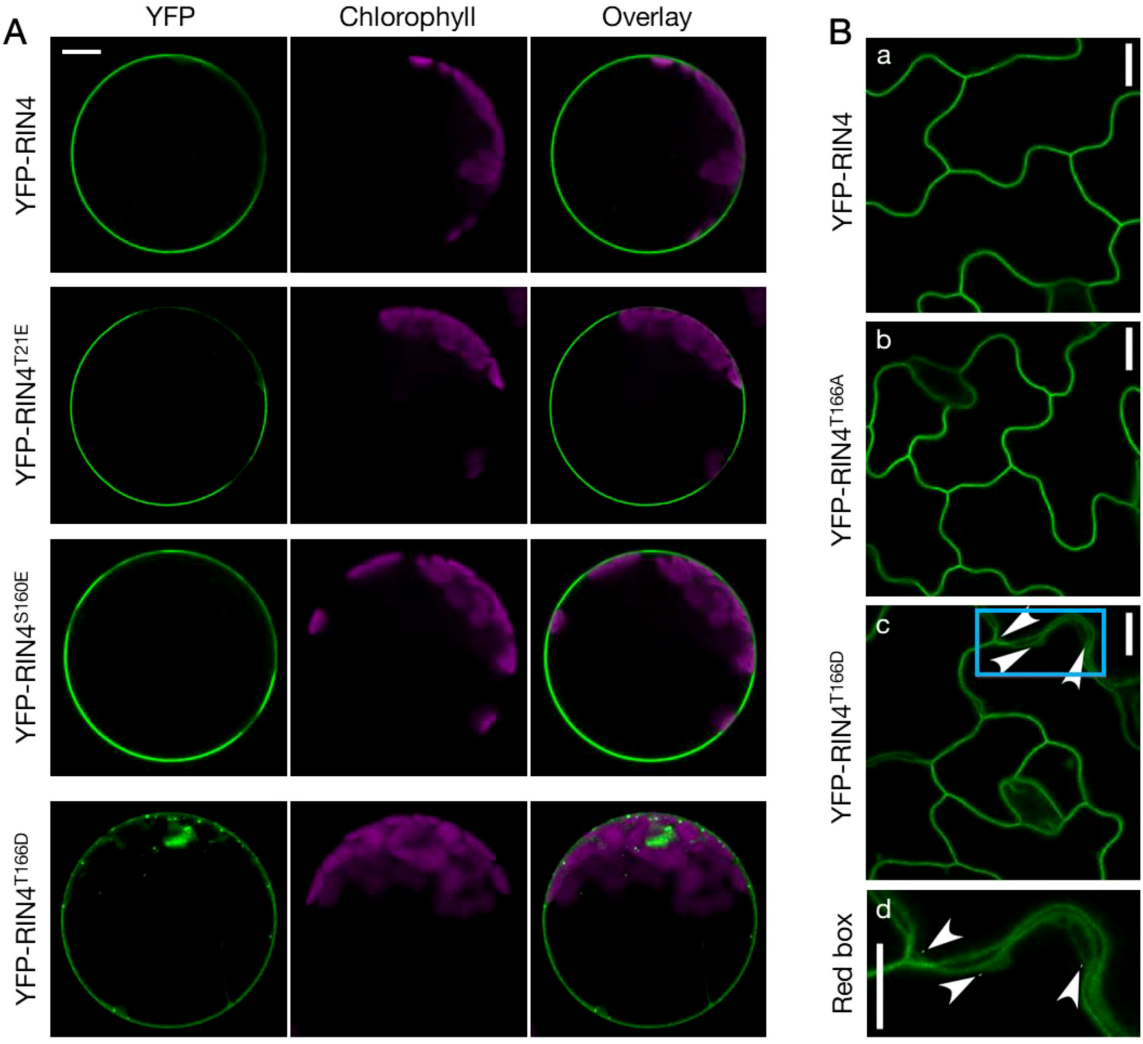
The phosphorylation status of RIN4 at Thr-166 modulates plasma membrane localization. **(A)** Transient expression of YFP-labelled RIN4 and its phosphomimetic variants (RIN4^T21E^, RIN4^S160E^, and RIN4^T166D^) in protoplasts extracted from Col-0. Punctate distributions of YFP-RIN4^T166D^ in protoplasts extracted from Col-0 were identified. Protoplasts were imaged using a Nikon C2 CLSM (40X oil-immersion objective, images at zoom 3). YFP fluorescence is pseudo-colored green, and chlorophyll is magenta. Images were captured for at least three biological replicates and there were at least 15 protoplasts of each line checked. Bar = 10 μm. **(B)** The T166D mutation of RIN4 alters proper plasma membrane localization in *Arabidopsis*. The subcellular localization of YFP fused RIN4 (a), RIN4^T166A^ (b), and RIN4^T166D^ (c) in epidermal cells. The arrowheads indicate where the protein does not show plasma-membrane localization. The bottom lane (d) shows the zoomed image in the sky-blue box. Transgenic lines expressing *35S promoter*::YFP-RIN4 isoforms in the *rpm1/rps2/rin4* genetic background were imaged with confocal and plants were 14-d-old. The images represent a single focal plane (60X oil-immersion objective, Nikon A1R-TIRF-STORM). Images were captured for at least three biological replicates. Bar = 10 μm.

Previous studies suggest that the suppression of PTI by secreted pathogen effector AvrRpt2 require the release of RIN4 from the PM (Takemoto and Jones, 2005; Afzal et al., 2011). To further evaluate this possible mechanism, we first examined the subcellular localization of a set of RIN4 phosphomimetic mutants – T21E, S160E, and T166D – the phosphorylation of which have been reported to be induced by the bacterial pathogen effector AvrB (Liu et al., 2011; Xu et al., 2017). As shown in **Figure 1A**, expression of RIN4^T166D^ resulted in the formation of punctate structures, whereas other single site phosphomimetic mutants of RIN4 displayed WT PM localization. This aberrant subcellular localization of RIN4^T166D^ was further confirmed in *Arabidopsis* transgenic lines producing the YFP-RIN4^T166D^ chimeric protein (**Figure 1B**). In contrast, the phospho-dead RIN4 mutants, in which T21, S160, or T166 residues were substituted with alanine, showed proper PM localization when expressed in WT protoplasts (**Supplementary Figure S1A**). Since AvrB triggers RIN4 hyperphosphorylation at Thr21, Ser160, and Thr166 (Xu et al., 2017), we next examined the subcellular localization of double- and triple-site phosphomimetic mutants of RIN4. It was found that all the RIN4 multiple phospho-variants had disrupted PM localization (**Supplementary Figure S1B**).

Next, we investigated whether the RIN4^T166D^ variant restricts the activation of plant innate immunity. Transgenic plants expressing T7-tagged RIN4 variants in the *r124* (*rpm1/rps2/rin4*) genetic background were generated (Belkhadir et al., 2004), and the flg22-priming assay was performed to evaluate the function of T166 in the PTI response. We found that plants expressing the RIN4^T166D^ isoform supported higher bacterial growth as compared to those expressing WT RIN4 or the RIN4^T166A^ variant (**Supplementary Figure S2A**), which is supported by published data (Chung et al., 2014). We further evaluated the activation of plant basal defense responses in these transgenic lines using the mutant bacteria *Pst* DC3000 *hrcC^-^*. As shown in **Supplementary Figure S2B**, plants expressing the RIN4^T166D^ variant allowed more bacterial growth than those expressing WT RIN4 or the phospho-dead variant RIN4^T166A^. Meanwhile, the RIN4 isoforms were produced at similar levels in these transgenic lines (**Supplementary Figure S2C**). Taken together, our results suggest that the phospho-status of T166 plays a significant role in modulating host PTI response and shows gain-of-function phenotype.

### 3.2 Phosphorylation at RIN4^T166^ leads to Golgi localization of this protein

**Figure 2:**
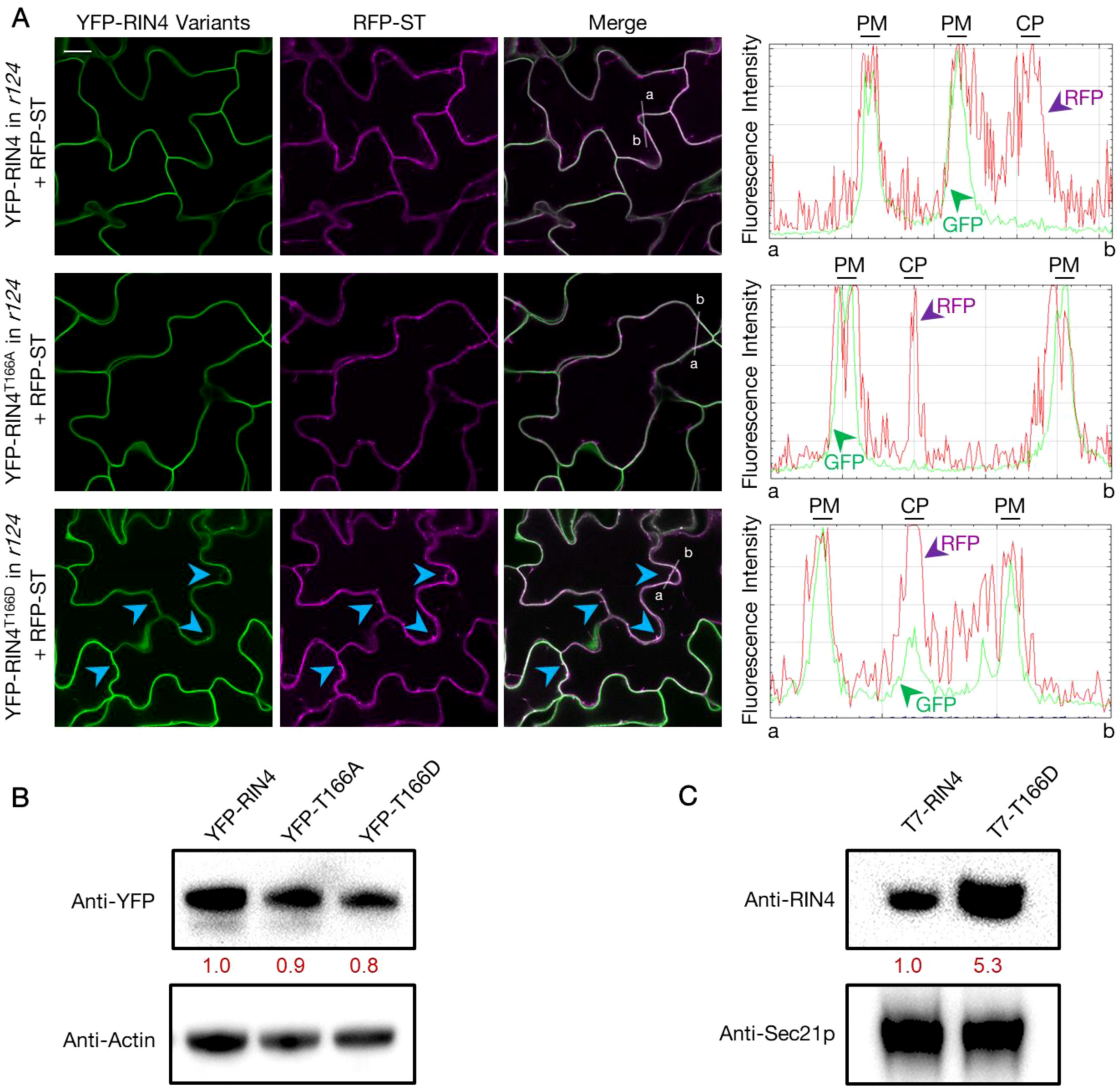
YFP-RIN4^T166D^ co-localizes with the Golgi apparatus marker. **(A)** Fluorescence images showing the subcellular localization of YFP fused RIN4 variants and RFP fused Golgi marker ST (RFP-ST) in the rpm1/rps2/rin4 genetic background. The panels from left to right display fluorescence images that showing YFP labelled RIN4 variants, RFP labelled ST, overlay fluorescence of YFP and RFP, and fluorescence intensity graphs which show YFP (green) and RFP (magenta) signals measured along the white line from a to b separately. The arrowheads indicate where the RIN4^T166D^ protein co-localizes with Golgi marker. The images represent a single focal plane (40X oil-immersion objective, Nikon A1R-TIRF-STORM). The 14-d-old plants were used. Images were captured for at least three biological replicates and there were at least 5 plants of each line checked. Bar = 20 μm. PM, plasma membrane; CP, cytoplasm. **(B)** Specific YFP-RIN4 protein detected in the total protein extracts of transgenic lines expressing YFP fused RIN4 isoforms (RIN4, RIN4^T166D^ or RIN4^T166A^) in the rpm1/rps2/rin4 genetic background. Immunoblotting analysis was performed with an anti-GFP antibody produced in rabbit (Sigma, SAB4301138). Plant actin was used as control. The relative quantities of the protein levels of RIN4 variants were calculated by using Fiji and indicated with red values. YFP-RIN4= *rpm1/rps2/rin4* expressing *35S::*YFP-RIN4 and *35S::*RFP-ST. YFP-RIN4^T166A^ = *rpm1/rps2/rin4* expressing *35S::*YFP-RIN4^T166A^ and *35S::*RFP-ST. YFP-RIN4^T166D^ = *rpm1/rps2/rin4* expressing *35S::*YFP-RIN4^T166D^ and *35S::*RFP-ST. **(C)** Western blot showing the enhanced RIN4^T166D^ protein level in Golgi. Immunoblotting analysis was performed on Golgi extractions of transgenic lines expressing RIN4 native promoter driven T7 tagged RIN4 isoforms (RIN4, RIN4^T166D^) in *rpm1/rps2/rin4*. The 14-d-old plants were used. The protein level of RIN4 wild-type and phosphorylation mutant (RIN4^T166D^) were detected in Golgi isolation with an anti-RIN4 antibody (rabbit anti-RIN4 antibody, homemade, 1:2000). Anti-Sec21p (Agrisera, AS08 327) was used as protein marker. The relative quantities of the protein levels of RIN4 variants were calculated using Fiji and are indicated in red. *r124* = *rpm1/rps2/rin4* triple mutant. Experiments were repeated twice with similar results.

To determine the nature of the YFP-RIN4^T166D^ puncta, we next performed co-localization analysis by detecting the pavement cells from stable transgenic lines co-expressing YFP fused RIN4 variants and RFP fused markers in *r124* background. We found that the YFP-labelled RIN4 and RIN4^T166A^ show perfect colocalization with the PM marker PIP2A, while RIN4^T166D^ displays both PM and cytoplasmic localization (**Supplementary Figure S3**). Then the Golgi localization of the RIN4^T166D^ protein was confirmed by full co-localization with the RFP labelled sialyltransferase (RFP-ST) at the Golgi apparatus (**Figure 2A**). To determine whether the cellular distribution of YFP-RIN4^T166D^ was affected by protein degradation, total protein extracts were isolated from transgenic lines and evaluated using anti-GFP antibody produced in rabbit (Sigma, SAB4301138) by western blot. The result showed specific bands for YFP-RIN4 isoforms, confirming protein stability (**Figure 2B**). Additionally, we extracted Golgi from stable transgenic lines expressing RIN4 native promoter driven T7-fused RIN4 variants (e.g., RIN4, RIN4^T166D^) and evaluated protein levels using anti-RIN4 antibody. As shown in **Figure 2C**, the protein level of RIN4^T166D^ was notably higher in Golgi compared to the WT RIN4 protein, which is in line with the observations of **Figure 2A**. These results indicated that the phosphorylation of RIN4 on T166 residue might block the transport of RIN4 to the PM, which induced protein aggregation in Golgi.

### 3.3 PM-localization of RIN4 requires actin cytoskeleton- and Exo70B1-dependent exocytosis

**Figure 3:**
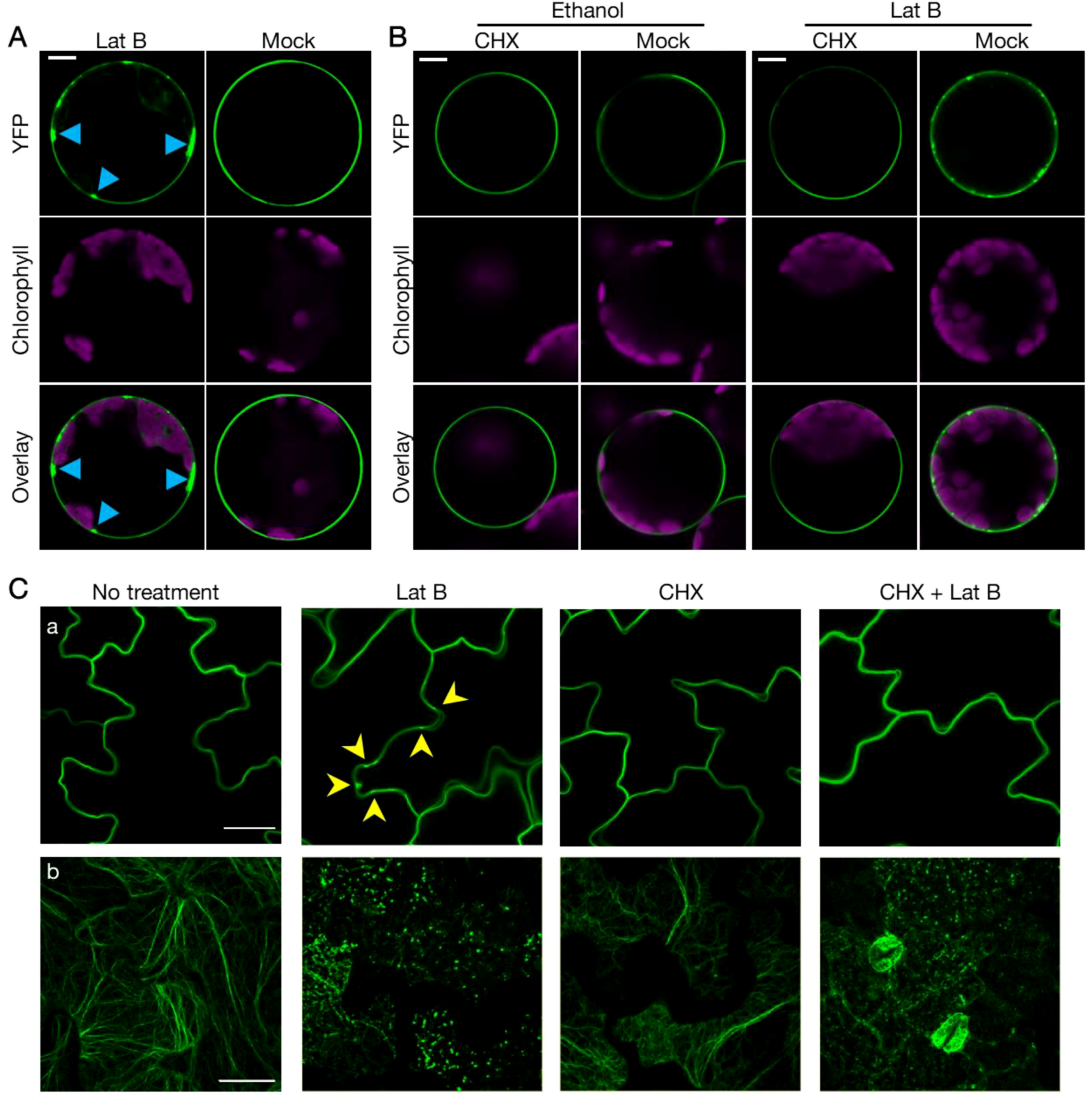
Proper localization of RIN4 requires exocytosis. **(A)** Proper subcellular localization of YFP-RIN4 was disrupted after Lat B treatment. 35S::YFP-RIN4 was transiently expressed in protoplasts extracted from WT Col-0. Upper right images show the proper plasma membrane localization of RIN4. After being treated with 0.1 μM Lat B for 30 min, the distribution of YFP-RIN4 showed puncta structures (upper left image, indicated by sky blue arrowheads). Protoplasts were imaged using a Nikon C2 CLSM (40X oil-immersion objective, images at zoom 3). YFP fluorescence is pseudo-colored green, and chlorophyll is magenta. Images were captured for at least three biological replicates. Bar = 10 μm. **(B)** CHX treatment complements the punctate distribution of YFP-RIN4 induced by LatB treatment. Protoplasts extracted from WT Col-0 were treated with 100 μg/mL cycloheximide (CHX) or 0.7% ethanol for 40 minutes. Following treatment, protoplasts were treated with 0.7% ethanol or 0.1 μM LatB for additional 30 minutes. Protoplasts were imaged using a Nikon C2 CLSM (40X oil-immersion objective, images at zoom 3). YFP fluorescence is indicated in green and chlorophyll autofluorescence is indicated in magenta. Images were captured for at least three biological replicates. Bar = 10 μm. **(C)** The proper localization of RIN4 requires exocytosis. Confocal laser scanning micrograph (CLSM) of epidermal cells from transgenic plants expressing 35S::YFP-RIN4 in WT Col-0 background **(a)** or the actin marker 35S::GFP-fABD2 in WT Col-0 background **(b)** under different chemical treatment. Following treatment of plants with 10 μM latrunculin-B (Lat B) for 15 min, the distribution of YFP-RIN4 showed puncta localization patterns (indicated with arrowheads), while pretreatment with cycloheximide (CHX) inhibited the Lat B-induced mis-localization of YFP-RIN4 **(a)**. The organization of actin cytoskeleton labelled with GFP-fABD2 was disrupted following Lat B treatment in samples following mock or CHX treatment **(b)**. Leaf discs were imaged using a Nikon C2 CLSM (60X oil-immersion objective). Three-dimensional reconstructions of confocal z-stacks are shown. 14-day-old seedlings were pretreated with or without 50 μM CHX for 2 hours, after which, LatB was added at a final concentration of 10 μM. YFP and GFP fluorescence are shown green. Experiments were performed in three independent biological replicates. Bar = 25 μm.

According to previous studies, there are two potential pathways through which RIN4 might localize to the PM. One is exocytosis, which transports protein to the PM after synthesis (Bassham and Raikhel, 1996; Ponnambalam and Baldwin, 2003; Chia and Gleeson, 2014). The other is protein endocytic recycling, where plasma membrane-localized protein enters cytoplasmic through endocytosis and then re-localizes to the PM (Grant and Donaldson, 2009). As actin has been linked in multiple ways with exocytosis and endocytosis, we hypothesized that cytoskeletal actin plays a role in mediating RIN4 PM localization (Chakrabarti et al., 2021). To test this hypothesis, we treated mesophyll protoplasts that were transiently expressing YFP-RIN4 with the actin antagonist LatB and then evaluated the subcellular localization of RIN4. As shown in **Figure 3A**, we observed the rapid formation of puncta in proximity to the PM upon treatment, suggesting that disruption of the host actin cytoskeleton negatively impacts membrane trafficking of RIN4 and leads to aggregation of the YFP-RIN4 protein (**Figure 3A**). When protoplasts are pretreated with the protein biosynthesis inhibitor cycloheximide (CHX), we hypothesized that the PM localization of RIN4 would follow the endocytic recycling pathway, as noted above, and the punctate structure(s) would be detected following LatB application. Interestingly, the impact of LatB on RIN4 location was eliminated when protoplasts were pretreated with CHX (**Figure 3B**). These observations demonstrate that PM localization of RIN4 does not rely on endocytic recycling, but rather depends on exocytosis. We then employed transgenic lines stably expressing YFP-RIN4 and observed that the mis-localization of RIN4 protein induced by LatB was also abolished by CHX pretreatment (**Figure 3C**). Taken together, our data suggests that membrane tethering of RIN4 relies on actin cytoskeleton-involved exocytosis.

To further investigate the mechanisms(s) underpinning this activity, we next asked how the function of the phosphomimic derivative RIN4^T166D^ intersects with the localization-dependent function of RIN4. As a first step, we asked if the exocytosis pathway contributes to PM localization of RIN4; this line of investigation is based on previous results demonstrating that RIN4 co-localizes with Exo70B1 in N. benthamiana (Sabol et al., 2017). After confirming the interaction using a split-YFP approach in N. benthamiana (**Supplementary Figure S4A**), we used a Y2H approach to demonstrate that both phosphomimetic mutants RIN4^T166D^ and RIN4^T21E/S160E/T166D^ abolished the interaction between RIN4 and Exo70B1 (**Supplementary Figure S4B**). Taken together, these data support the hypothesis that altered subcellular localization of RIN4^T166D^ (**Figure 1**) and the RIN4^T21E/S160E/T166D^ triple mutant (**Supplementary Figure S1B**) is likely due to a loss in the interaction between RIN4 and Exo70B1.

**Figure 4:**
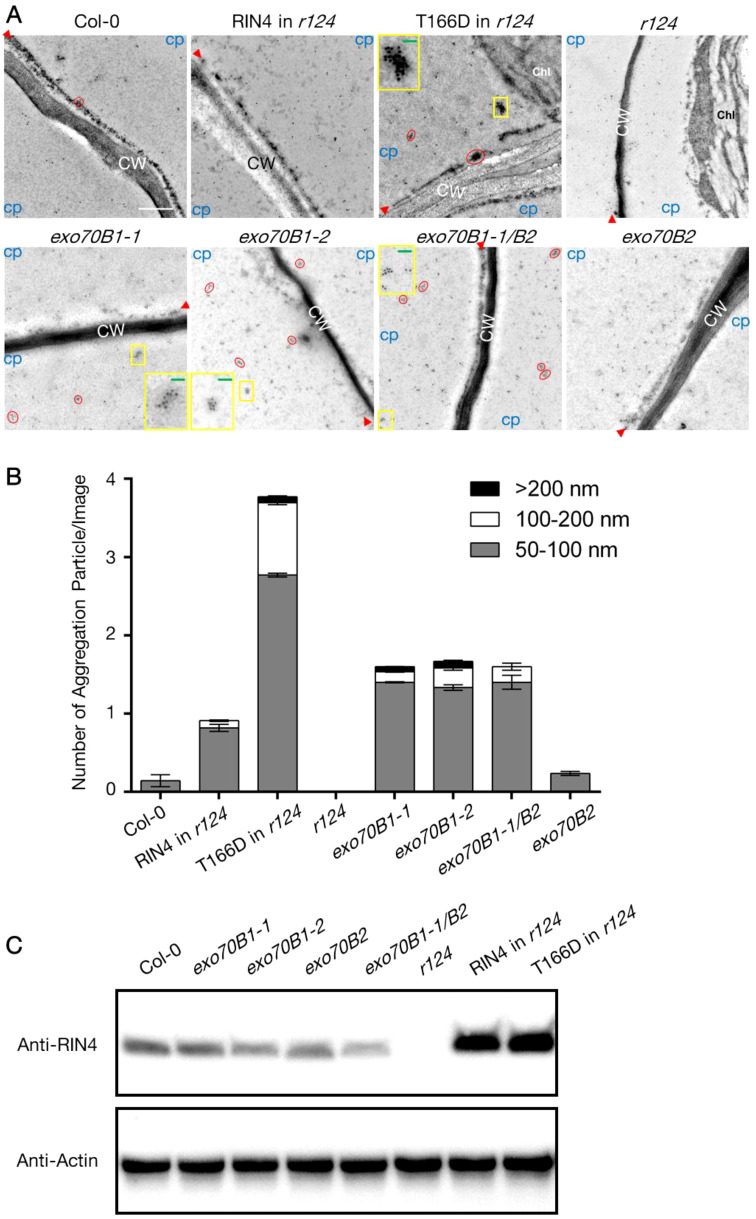
The altered subcellular localization of RIN4 in *exo70B1* mutants. **(A)** Subcellular localization of RIN4 depends on its phosphorylation status and Exo70B1. Immunogold labeling of RIN4 from samples of WT Col-0, T7-RIN4/*r124*, T7-RIN4^T166D^/*r124*, *r124*, *exo70B1-1*, *exo70B1-2*, *exo70B1-1*/*exo70B2*, and *exo70B2*. Red circles show accumulation of RIN4 in aggregates. CW, cell wall; Chl, chloroplast; CP, cytoplasmic area. Protein aggregations in the yellow boxes are zoomed in images and presented in the corner of individual image. Red arrowheads indicate the position of plasma membrane. Experiments were performed for three biological replicates (n ≥ 5/line). *r124*, *rpm1/rps2/rin4* triple mutant; RIN4 in *r124*, *gRIN4p::*T7-RIN4 in *rpm1/rps2/rin4* background; RIN4^T166D^ in *r124*, *gRIN4p::*T7-RIN4^T166D^ in *rpm1/rps2/rin4* background. Bar = 500 nm. Green bars in the zoomed-in image equal to 200 nm. **(B)** The proportion of different types of RIN4 variants aggregation particle in pavement cells. The values represent the average mean ± SEM. Statistical significance was determined by two-way ANOVA. In the 50-100 Diameter group, the P-values of RIN4 in *r124*, RIN4^T166D^ in *r124*, *exo70B1-1*, *exo70B1-2*, and *exo70B1-1*/*exo70B2* compared to Col-0 were < 0.0001; the P-value of *r124* compared to Col-0 was 0.0001; the P-values of RIN4^T166D^ in *r124*, *exo70B1-1*, *exo70B1-2*, *exo70B1-1*/*exo70B2*, and *exo70B2* compared to RIN4 in *r124* were < 0.0001; no significate difference was detected between Col-0 and *exo70B2*; the P-values of *exo70B1-1*, *exo70B1-2*, and *exo70B1-1*/*exo70B2* were insignificant. In the 100-200 nm group, the P-values of Col-0, RIN4 in *r124*, *r124, exo70B1-1*, *exo70B1-2*, *exo70B1-1*/*exo70B2*, and *exo70B2* compared to RIN4^T166D^ in *r124* were < 0.0001; P-values of Col-0, *r124*, and *exo70B2* compared to *exo70B1-1*/*exo70B2* were < 0.0001; the P-values of RIN4 in *r124*, *exo70B1-1*, *exo70B1-2* compared to *exo70B1-1*/*exo70B2* were insignificant; the P-values of RIN4 in *r124, r124, exo70B2* compared to Col-0 were insignificant. In the >200 nm group, the P-values of RIN4^T166D^ in *r124*, and *exo70B1-2* compared to *r124* were 0.0424 and 0.0248; the P-value of *exo70B2* compared to *exo70B1-2* is 0.0341; no significant difference was detected when compare P-values of any other two lines. The experiment was performed for three biological replicates (n ≥ 5/line). *r124*, *rpm1/rps2/rin4* triple mutant; RIN4 in *r124*, *gRIN4p::*T7-RIN4 in *rpm1/rps2/rin4* background; RIN4^T166D^ in *r124*, *gRIN4p::*T7-RIN4^T166D^ in *rpm1/rps2/rin4* background. **(C)** Detection of RIN4 protein level in the total protein extracts of lines that used for immunogold labelling assay in **(B)**. The anti-RIN4 antibody (rabbit anti-RIN4 antibody, homemade, 1:2000) was used to detect RIN4 protein. Plant actin was used as control. *r124*, *rpm1/rps2/rin4* triple mutant; RIN4 in *r124*, *gRIN4p::*T7-RIN4 in *rpm1/rps2/rin4* background; RIN4^T166D^ in *r124*, *gRIN4p::*T7-RIN4^T166D^ in *rpm1/rps2/rin4* background.

We then asked whether Exo70B1 was required for PM-localization of RIN4. To do this, we detected the natural distribution of RIN4 protein in exo70 mutants and RIN4 variants expression lines using immunogold labelling assay and collected the images with transmission electron microscope (**Figure 4**). The leaf-sections from 4-week-old plants were specific labeled with RIN4 antibody, and the results showed that the RIN4 displayed a similar distribution in WT Col-0, exo70B2, and 35S::YFP-RIN4 in *r124* where gold particles gave specific and clear labeling along the PM and non-specific labeling in the cytoplasm. However, in the transgenic line expressing the T166 phosphomimetic variant of RIN4, YFP-RIN4^T166D^, RIN4 was detected in aggregates (**Figure 4A**).

To quantify the protein aggregation level of each line, we first define a valuation standard as the shape of RIN4 (or RIN4^T166D^) aggregation is irregular (**Supplementary Figure S5**). Firstly, we drew lines between outermost nanoparticles of an aggregation and then got a closed graph. A line (named lineA) that links two furthest points was drawn and measured. After that, a rectangular coordinate system, whose y-axis parallels lineA and x-axis passes through the midpoint of lineA, was made. Two points where graph crossed the x-axis were named 1 and 1’, and the distance between these two points was measured (lineB). The average of lineA and lineB (Size=(“length lineA” + “length lineB”)/2) was calculated and the outputs were divided into 3 groups based on size: 50-100 nm, 100-200 nm, and >200 nm. **Figure 4B** shows the average number of each type per image and r124 expressing gRIN4p::T7-RIN4^T166D^ contains more RIN4^T166D^ aggregations, whose sizes were between 50-100 nm (2.77 ± 0.03) and 100-200 nm (0.92 ± 0.03), than other lines (**Figure 4B**). This result is consistent with our hypothesis that the phosphomimetic mutant RIN4^T166D^ induces RIN4 aggregation and blocks its tethering to the PM. Interestingly, mutants lacking Exo70B1 showed similar labeling patterns, with abundant RIN4 aggregations detected in the cytoplasm. It should also be noted that the RIN4 aggregates in exo70B1 knockout mutants bound RIN4 antibody less heavily than those of the RIN4^T166D^ transgenic line, which might because the RIN4^T166D^ was overexpressed in *r124* background so higher protein level leads to heavy aggregation (**Figure 4C**).

### 3.4 Genetic ablation of Exo70B1 compromises PTI response

**Figure 5:**
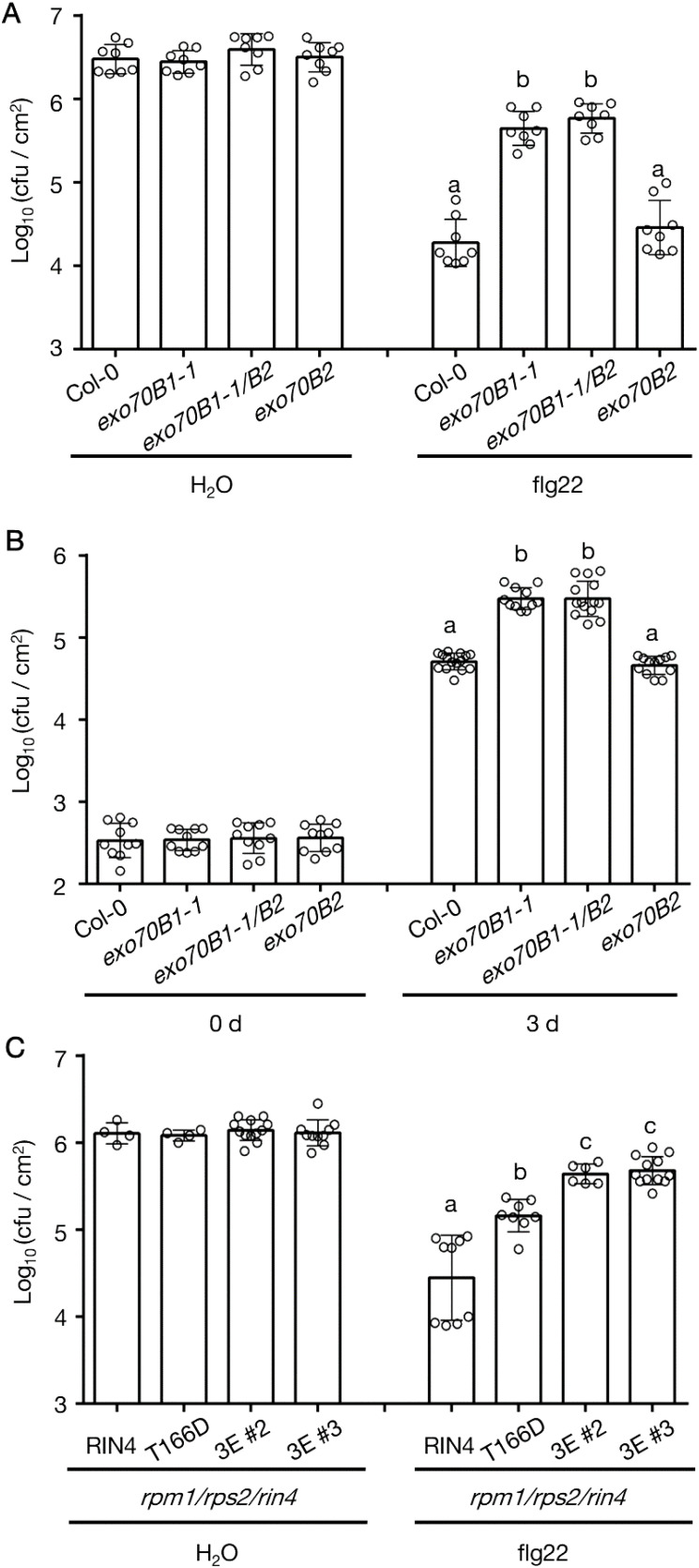
The PTI response in *exo70B1* phenocopies RIN4^T166D^ transgenic line. **(A)** The flg22-priming assay showing repressed PTI phenotype of exo70B1-1 and exo70B1-1/B2 (n = 8/genotype and treatment). Significance was determined by one-way ANOVA with Tukey-Kramer HSD with 95% confidence (Col-0 to exo70B1-1: P < 0.0001; to exo70B1-1/B2: P < 0.0001). Error bars represent SD. All experiments were repeated three times with similar results. **(B)** The loss of Exo70B1 shows enhanced susceptibility to Pst DC3000 hrcC^-^. Leaves were infiltrated with *Pst* DC3000 *hrcC^-^* at 1 x 10^6^ cfu/mL to induce PTI, and samples were collected at 0-day and 3-day post-inoculation (n ≥ 10/genotype and treatment). Significance was determined by one-way ANOVA with Tukey-Kramer HSD with 95% confidence (Col-0 to exo70B1-1: P < 0.0001; to exo70B1-1/B2: P < 0.0001). Error bars represent SD. All experiments were repeated three times with similar results. **(C)** The flg22-priming assay reveals enhanced bacterial growth in transgenic lines expressing RIN4^T21E/S160E/T166D^ in the r124 genetic background (n ≥ 4/genotype and treatment). Significance was determined by one-way ANOVA with Tukey-Kramer HSD with 95% confidence (RIN4 to T166D: P < 0.0001; to 3E (#2 or #3): P < 0.0001; T166D to 3E (#2 or #3): P < 0.02). Error bars represent SD. *r124*, *rpm1/rps2/rin4* triple mutant; 0 d, 0 day post infiltration; 3 d, 3 days post infiltration; RIN4, *35S* promoter-driven YFP-RIN4 in the r124 genetic background; T166D, *35S* promoter-driven YFP-RIN4^T166D^ in the r124 genetic background; 3E, *35S* promoter-driven RIN4^T21E/S160E/T166D^ in the r124 genetic background. All experiments were repeated three times with similar results. Different letters above flg22 treatment and day 3 bars (a, b, c) indicate statistically significant differences between the samples.

The PTI response was repressed in r124 plants that produce the RIN4^T166D^ mutant protein (**Supplementary Figure S2**). To continue this line of investigation, we pretreated exo70B mutants with flg22 followed by inoculation with the virulent pathogen Pst DC3000. Simultaneously, we infiltrated exo70B leaves with Pst DC3000 hrcC^-^ to evaluate induction of the PTI response. Both exo70B1-1 and exo70B1-1/exo70B2 plants were more susceptible to the bacteria, while exo70B2 showed comparable susceptibility to WT plants (**Figures 5A, B**). Moreover, an additional mutant allele exo70B1-2 phenocopied *exo70B1-1* in regard to their PTI responses (**Supplementary Figures S6A, B**). To address whether the increased susceptibility of *exo70B1* mutants was caused by enhanced expression of *RIN4*, we compared the transcription level of *RIN4* in *exo70B1*, *exo70B2*, and WT plants. It was found that the transcript abundances of RIN4 in the exo70 mutants were comparable to that in WT Col-0 (**Supplementary Figure S6C**). We next sought to determine whether the disease resistance was repressed further by expressing the triple phospho-variant in r124, since the disruption of RIN4 PM localization was enhanced when expressing the RIN4^T21E/S160E/T166D^ mutant (**Supplementary Figure S1B**). As shown in **Figure 5C**, susceptibility of the two transgenic lines expressing RIN4^T21E/S160E/T166D^ was comparable to that of exo70B1 mutants. The data showed that flg22-induced resistance is reduced in RIN4^T166D^ and RIN4^T21E/S160E/T166D^ expression lines and *exo70B1* genetic mutants.

**Figure 6:**
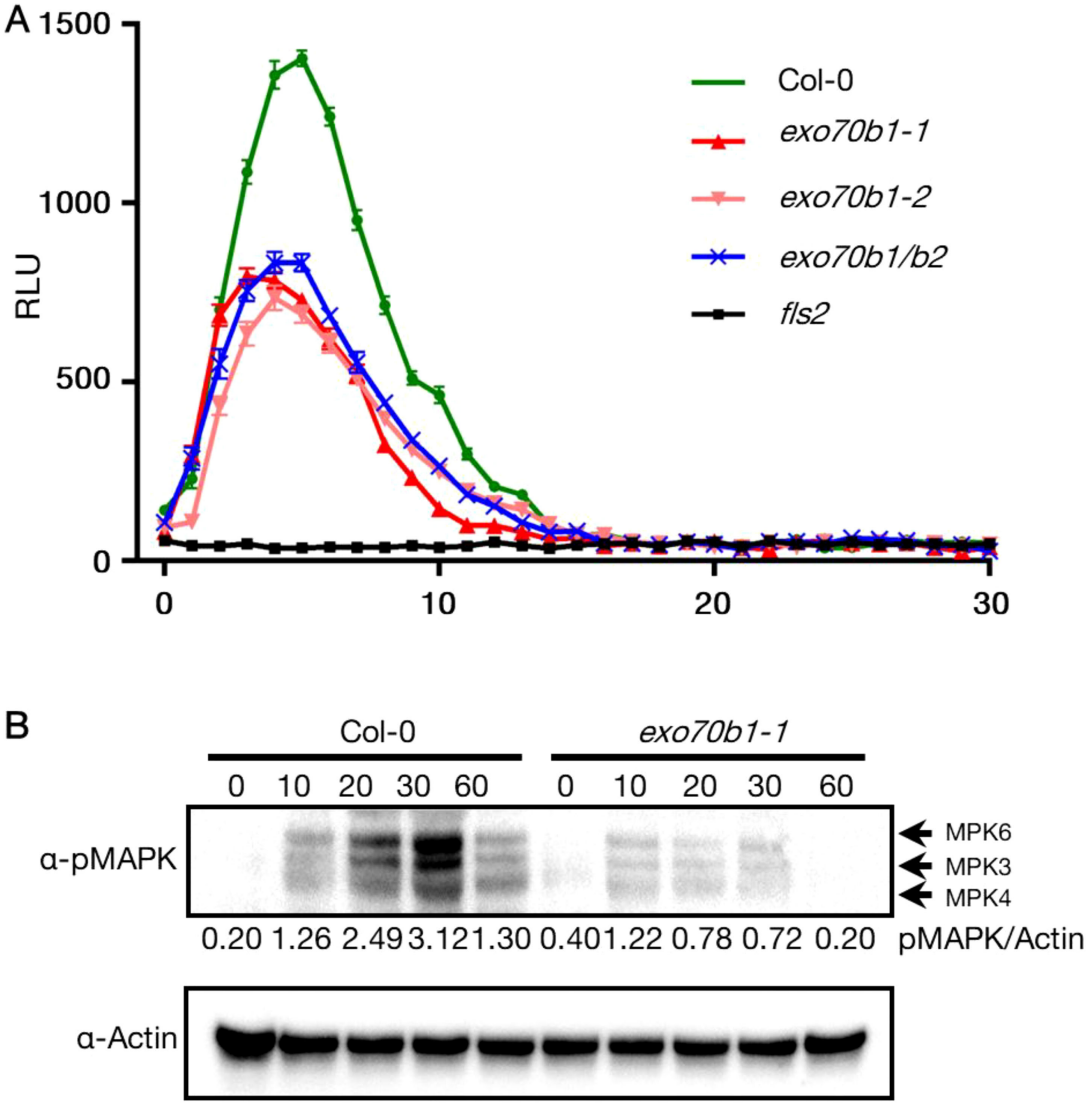
Reduced MAPK activation and ROS burst in *exo70b1*. **(A)** Reduced ROS burst in *exo70b1-1*, *exo70b1-2*, and *exo70b1/b2*. The ROS burst in Col-0 was positive control and in *fls2* was negative control. Luminol assays were performed after 100 nM flg22-treatment. Data were collected from at least 10 leaf discs (n > 10) for each genotype with four independent replicates. Error bars represent SE. All experiments were repeated three times with similar results. **(B)** Reduced MAPK activation in *exo70b1-1*. Fully expanded leaves of 4-week-old plants were infiltrated with 1 µM flg22, and samples were collected at 0, 10, 20, 30, and 60 min after infiltration. MAPK activity was detected using immunoblots with α- p44/42 MAPK (Erk1/2, cell signaling). The relative MAPK protein levels were normalized to the corresponding levels of actin and are shown below each lane. The assay was repeated three times, and representative blots are shown. All experiments were repeated three times with similar results.

Given that rin4 plants are reduced in the flg22-induced ROS burst (Lee et al., 2015), we analyzed the flg22-triggered ROS burst in WT Col-0, *exo70B1-1*, exo70B1-2, *exo70B1-1*/exo70B2, and *fls2*. The *fls2* mutant was used as negative control, and WT Col-0 was the positive control. Upon flg22 treatment, similar observation of reduced ROS level was made in *exo70B1-1*, exo70B1-2, and *exo70B1-1*/exo70B2 mutants (**Figure 6A**), which is consistent with previous report (Stegmann et al., 2012). Since PTI response also includes the activation of mitogen-activated protein (MAP) kinases, we tested MAPK activity levels in *exo70B1-1* and WT Col-0 at 0-, 10-,20-, 30-, 60-min upon flg22 infiltration. MAPK activation was also reduced in *exo70B1-1* plants when compared with WT Col-0 (**Figure 6B**). Taken together, we posit that PTI responses are dampened.

### 3.5 Repressed PTI response in exo70B1 is not complemented by overexpressing RIN4^T166D^

**Figure 7:**
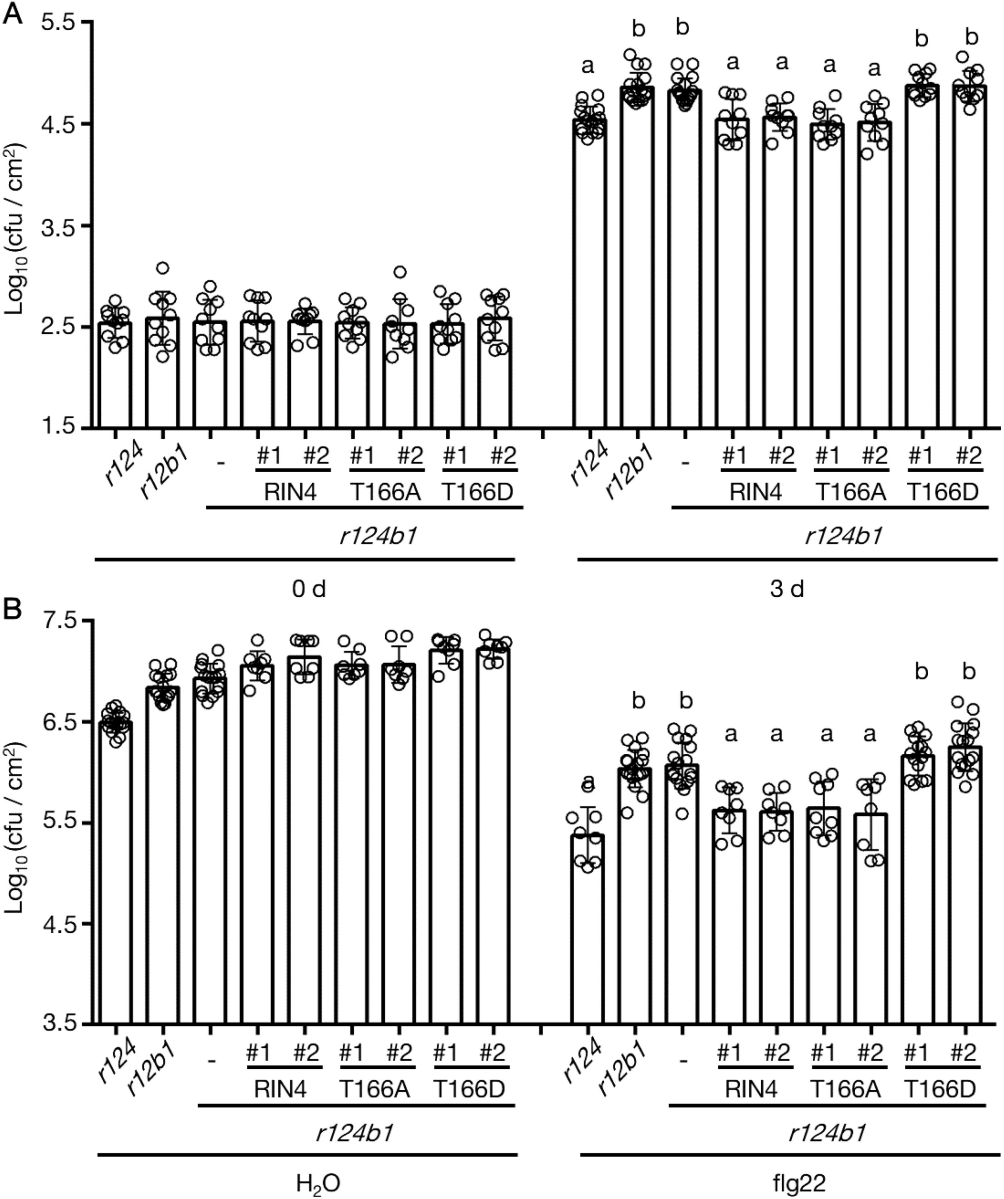
The reduced PTI response which was caused by *Exo70B1* knockout was not complemented by RIN4^T166D^. **(A)** Leaves were infiltrated with *Pst* DC3000 *hrcC^-^* at 1 x 10^6^ cfu/mL to induce PTI, and samples were collected at 0-day and 3-days post-inoculation (n ≥ 10/genotype and treatment). Significance was determined by one-way ANOVA with Tukey-Kramer HSD with 95% confidence (*r124* to *r12b1*: P < 0.0001; to *r124b1*: P < 0.0001; to T166D #1 in *r124b1*: P < 0.0001; to T166D #2 in *r124b1*: P < 0.0001). Error bars represent SD. RIN4, *35S* promoter-driven YFP-RIN4 in the r124b1 genetic background; T166A, *35S* promoter-driven YFP-RIN4^T166A^ in the r124b1 genetic background; T166D, *35S* promoter-driven YFP-RIN4^T166D^ in the r124b1 genetic background. *r124* = *rpm1/rps2/rin4* triple mutant, *r124b1* = *rpm1/rps2/rin4/exo70B1-1* quadruple mutant, *r12b1* = *rpm1/rps2/exo70B1-1* triple mutant. All experiments were repeated three times with similar results. Different letters above day3 bars (a, b) indicate statistically significant differences between the samples. **(B)** The flg22-priming assay showing enhanced bacterial growth in *rpm1/rps2/rin4/exo70B1-1* and *rpm1/rps2/exo70B1-1*. The expression of RIN4^T166D^ in *rpm1/rps2/rin4/exo70B1-1* background did not complement the reduced PTI (n ≥ 8/genotype and treatment). Significance was determined by one-way ANOVA with Tukey-Kramer HSD with 95% confidence (*r124* to *r12b1*: P < 0.0001; to *r124b1*: P < 0.0001; to T166D #1 in *r124b1*: P < 0.0001; to T166D #2 in *r124b1*: P < 0.0001). Error bars represent SD. RIN4, *35S* promoter-driven YFP-RIN4 in the r124b1 genetic background; 0 d, 0 day post infiltration; 3 d, 3 days post infiltration; T166A, *35S* promoter-driven YFP-RIN4^T166A^ in the r124b1 genetic background; T166D, *35S* promoter-driven YFP-RIN4^T166D^ in the r124b1 genetic background. *r124* = *rpm1/rps2/rin4* triple mutant, *r124b1* = *rpm1/rps2/rin4/exo70B1-1* quadruple mutant, *r12b1* = *rpm1/rps2/exo70B1-1* triple mutant. All experiments were repeated three times with similar results. Different letters above flg22 treatment bars (a, b, c) indicate statistically significant differences between the samples.

To confirm the enhanced susceptibility of *exo70B1* mutants to Pseudomonas syringae pv. tomato is related to RIN4 phosphorylation at T166 site, the stable transgenic lines overexpressing RIN4 variants in *rpm1-3/rps2-101c/rin4/exo70B1-1* (*r124b1*) mutant background were prepared and checked with the PTI response and basal defense. By choosing *r124b1* quadruple mutant, not *exo70B1* mutant, as transgenic background, we eliminate the side effect of ETI induced by RIN4 variants. Compared to *r124*, *rpm1-3/rps2-101c/exo70B1-1* (*r12b1*) was more susceptible to bacteria like *rpm1-3/rps2-101c/rin4/exo70B1-1* (r124b1), which indicates RIN4 and Exo70B1 are in the same PTI regulation pathway and Exo70B1 has a bigger effect on PTI than RIN4. Interestingly, overexpressing of RIN4 or RIN4^T166A^ in the *r124b1* background reduced pathogen growth compared to *r12b1*, while RIN4^T166D^-expression did not (**Figure 7**). This not only proves RIN4 plays a role downstream of Exo70B1 and proper PM localization of RIN4 requires Exo70B1, but also indicates that the gain-of-function of phospho-RIN4^T166D^ requires Exo70B1 even though their interaction is disrupted by this RIN4 mutation. Additionally, we noticed that the phenotype of *r124b1* was complemented in *35S*::YFP-RIN4/*r124b1* (**Figure 7**). This may be due to the constitutive expression of RIN4 leading to a higher protein level than in WT Col-0, with some of proteins localized to the PM. Taken together, these analyses support the model that the PM-localization of RIN4, which is mediated by Exo70B1, plays a role in PTI response, and the phosphorylation of RIN4 at T166 site represses PTI by inhibiting RIN4-Exo70B1 interaction.

## 4 Discussion

**Figure 8:**
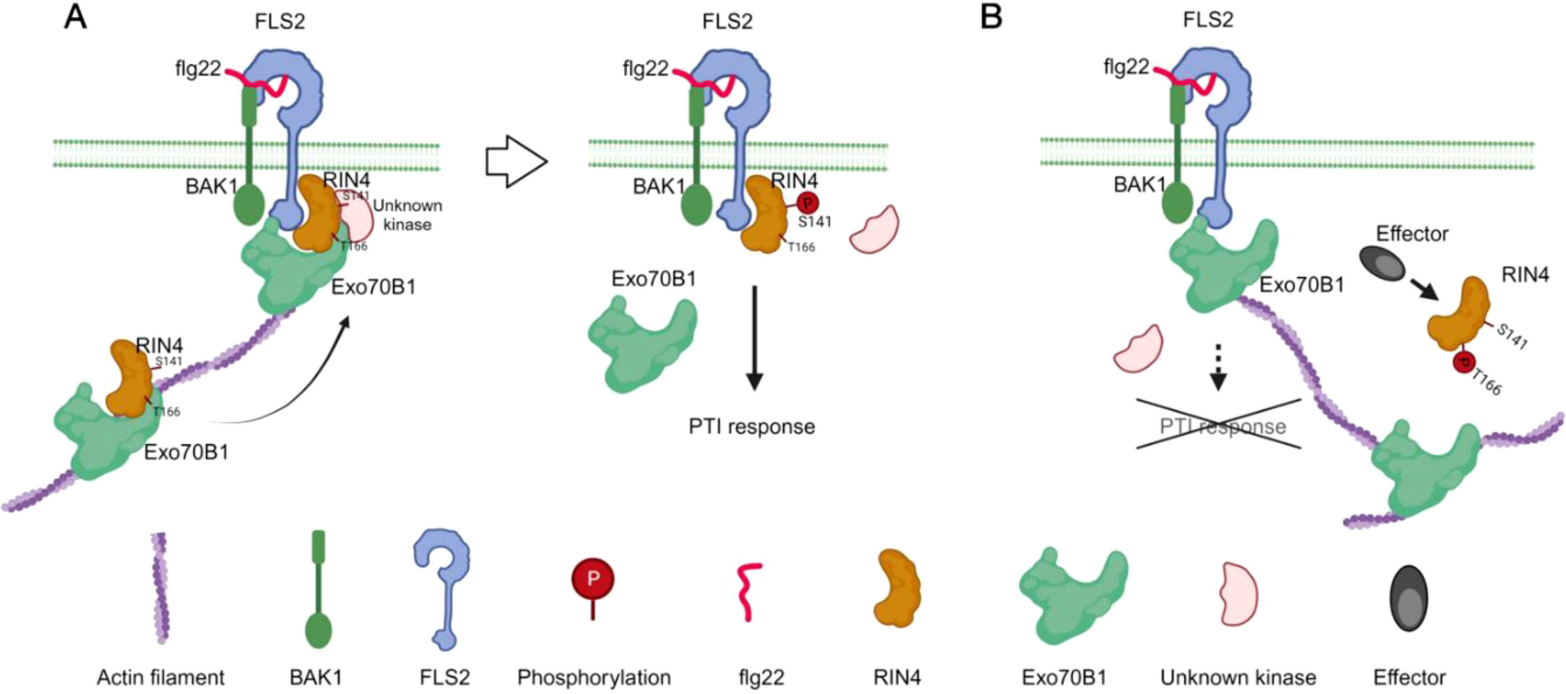
Schematic model of the function of the RIN4-Exo70B1 in plant immunity. RIN4 is trafficked to the plasma membrane along actin filaments, a process that requires the interaction between Exo70B1 and RIN4. Flg22-elicited phosphorylation of RIN4 at S141 site triggers PTI response **(A)**. When RIN4 is phosphorylated at T166 upon effectors injection, the interaction between RIN4 and Exo70B1 is abolished. Then the membrane tethering process of RIN4 is blocked and results in reduced PTI responses **(B)**.

RIN4’s role as a negative regulator of plant immunity has become well established, linking pathogen virulence with the regulation of both PAMP-triggered immunity (PTI) and effector-triggered immunity (ETI) (Kim et al., 2005). Recent studies have highlighted the significance of the phosphorylation “switch” on RIN4 (Chung et al., 2014), advancing our understanding of the signaling pathways that connect PTI, particularly via Ser-141, to ETI induced by AvrB and AvrRpm1 through Thr-166. In this study, we explored how phosphorylation of RIN4—especially at Thr-166—affects its spatial distribution. Our findings offer new insights into the mechanisms controlling RIN4’s subcellular localization in plant immunity, emphasizing the role of Exo70B1-dependent vesicle trafficking in RIN4-mediated immune signaling (**Figure 8**).

### 4.1 PM localization of RIN4 is abolished by its phosphorylation at T166

The function of RIN4 in plant immunity is regulated by phosphorylation, which is induced by both virulence factors of pathogens and host enzymes. Phosphorylation at specific residues of RIN4 has different effects on the activation or suppression of PTI signaling. It’s well known that flg22-induced phosphorylation of RIN4 at S141 by host BIK1 and PBS1-like 1 kinases enhances PTI signaling (Lu et al., 2010; Zhang et al., 2010; Chung et al., 2014; Li et al., 2014). However, the effect of S141 phosphorylation is suppressed by phosphorylation of T166, which leads to PTI repression (Chung et al., 2014). Phosphorylation at RIN4^T166^, induced by AvrRpm1 and AvrB, requires CDG1 (CONSTITUTIVE DIFFERENTIAL GROWTH1) and RIPK (RPM1-induced protein kinase) (Chung et al., 2011; Liu et al., 2011; Yang et al., 2021). Besides this, RIN4 is hyperphosphorylated by RIPK at T21, S160, and T166 and then activates RPM1-mediated ETI (Liu et al., 2011; Lee et al., 2015; Xu et al., 2017). To date, studies on phosphorylation of RIN4 have been focusing on the molecular mechanisms underlying pathological phenotype, while little attention has been paid to the cellular biological regulation. It is noteworthy that a previous study claimed that the nonmembrane-tethered RIN4 derivatives suppress PTI more potently, but no further mechanism was explored (Afzal et al., 2011). In our work, we demonstrated that the subcellular localization of RIN4 is altered by phosphorylation at T166, and/or at two residues of T21, S160, and T166 residues (**Figure 1**; **Supplementary Figure S1**). Transgenic lines expressing RIN4^T166D^ displayed gain-of-function PTI phenotypes (**Supplementary Figure S2**), suggesting that proper PM localization of RIN4 is required for plant immunity. Then, the question raised by our work is whether pathogen or PAMP treatment alters the PM localization of RIN4. To investigate this, we infiltrated leaves of stable transgenic plants expressing YFP-RIN4 with H_2_O, AvrB, and AvrPphB and detected the subcellular localization of RIN4 at 1 h and 3 h post-treatment. We found that both AvrB and AvrPphB treatments led to altered localization of RIN4, and no significant difference was detected between the two treatments.

### 4.2 The interaction between RIN4 and Exo70B1 is required for PTI

The exocyst complex, a conserved complex found in yeast, mammals, and plants, plays a role in protein delivery to the PM (Liu and Guo, 2012). The exocyst complex consists of eight subunits: Sec3, Sec5, Sec6, Sec8, Sec10, Sec15, Exo70, and Exo84 (Elias et al., 2003; Hsu et al., 2004; Hala et al., 2008; Cvrckova et al., 2012; Martin-Urdiroz et al., 2016; Mei and Guo, 2018; Mei et al., 2018; Zhu et al., 2018). In Arabidopsis, each exocyst subunit has gene duplications (Cvrckova et al., 2012), and in the case of Exo70, it has evolved into 23 paralogs (Zarsky et al., 2020). These isoforms possess specific expression patterns and biological functions that further define and differentiate the distinct functions of exocyst (Saeed et al., 2019; Pecenkova et al., 2020). Given that RIN4 interacts with Exo70B1 and RIN4 pT166 eliminates the RIN4/Exo70B1 association (Sabol et al., 2017; Redditt et al., 2019; Yuen et al., 2023), we first confirmed this conclusion by performing BiFC and yeast-two-hybrid (**Supplementary Figure S4**). Furthermore, the PM localization of RIN4 was also altered in exo70B1 mutants and in RIN4^T166D^ stable transgenic line, coinciding with suppressed PTI responses, including ROS burst and MAPK activation (**Figures 4**–**6**; **Supplementary Figure S6**). These results support the hypothesis that the interaction between Exo70B1 and RIN4 is required for PM localization of RIN4 as well as the activation of the RIN4-dependent PTI response.

In support of the above, we observed higher bacterial growth in exo70b1 compared to the RIN4^T166D^ transgenic line. Interestingly, in planta bacterial growth loads were comparable to those observed in the RIN4^T21E/S160E/T166D^ line (**Figure 5**). This indicates that the repression of PTI by phosphorylated RIN4 might depend on the interaction affinity between RIN4 and Exo70B1. It should be noted that our observations differ from previous studies (Zhao et al., 2015; Liu et al., 2017), and it is worth pointing out that the repressed PTI responses observed in exo70B1-1 and exo70B1-2 are contrary to the previous conclusions which reported enhanced disease resistance in exo70B1-3.

Based on our observation that all three exo70B1 knockout mutants exhibited HR-like cell death and that both exo70B1-1 and exo70B1-2 showed reduced resistance against virulent Pst and Pst hrcC- (Kulich et al., 2013; Stegmann et al., 2013), we propose that additional genomic mutations may be present and warrant further investigation. Previous research reported that exo70B1-3 had lower FLS2 accumulation at the PM compared to WT Col-0 (Wang et al., 2020). As FLS2 is a receptor kinase for flg22, a decrease in its PM levels correlates with reduced flg22 responses (Wang et al., 2014; Orosa et al., 2018). However, to determine if the reduced FLS2 levels in exo70B1-3 are due to the absence of Exo70B1 or other genomic mutations, it is essential to investigate FLS2 levels at the PM in exo70B1-1 and exo70B1-2 as well. Moreover, assessing pathogen growth in RIN4^T166A^/r124b1 compared to r12 could provide insights. If the repressed PTI response in exo70B1-1 and exo70B1-2 results from reduced FLS2 rather than solely disrupted RIN4-Exo70B1 interaction, RIN4^T166A^/r124b1 should phenocopy r12. Notably, phosphorylation at Thr-166 on RIN4 enhances its interaction with EXO70E2, another exocyst subunit involved in flg22-induced callose deposition (Redditt et al., 2019). This interaction inhibits callose deposition in the RIN4^T166D^ variant, suggesting that EXO70E2 may contribute to PTI suppression in stable transgenic lines expressing RIN4^T166D^.

As shown in **Figure 7**, we observed that *r12b1* supported similar pathogen growth level as r124b1, and that transgenic expression of RIN4^T166D^ in the *r124b1* background did not complement the enhanced bacterial growth of *r124b1*. However, expression of WT RIN4 and RIN4^T166A^ did complement (**Figure 7**). This data provides further evidence that the proper PM localization of RIN4 is required for the execution of Exo70B1-related PTI responses, downstream of Exo70B1. Thus, we propose that RIN4 plays a critical role in the spatiotemporal control of complex immune signaling dynamics required for membrane trafficking and plant secretory pathways. In addition, the higher susceptibility of r12b1 to Pst hrcC^-^ and DC3000 than *r124*, indicating that Exo70B1 affects more factors in PTI process besides RIN4, requires further exploration.

In summary, the data presented here provide new insights into the mechanisms connecting effector-induced RIN4^T166 phosphorylation with PTI suppression. A central aspect of this mechanism involves the precise modulation of the interaction between RIN4 and Exo70B1. This study enhances our understanding of the spatiotemporal coordination between RIN4 and exocytosis in plant-pathogen interactions, shedding light on both conserved and unique functions of RIN4 in plant immunity.

## Data Availability

The original contributions presented in the study are included in the article/**Supplementary Material**, further inquiries can be directed to the corresponding author/s. Sequence data from this article can be found in the Arabidopsis TAIR database (http://www.arabidopsis.org) Under the following accession numbers: AT3g25070 (RIN4), AT5g58430 (Exo70B1), AT1g07000 (Exo70B2), and At3g02780 (IPP2).
