# Supplementary material for: Subcellular spatial regulation of immunity-induced phosphorylation of RIN4 links PAMP-triggered immunity to Exo70B1
Source: Front Plant Sci. 2024 Dec 13;15:1473944. doi: 10.3389/fpls.2024.1473944 (PMC11681384; doi:10.3389/fpls.2024.1473944)
Supplement: Supplementary file 1 [file DataSheet1.docx]

**Supplementary Materials and Figures**

**Table S1. List of the DNA primers used in this study.**

| **Primer name** | | **DNA Primer Sequence (5´ to 3´)** |
| --- | --- | --- |
| RPM1-F  RPM1-R  RPS2-F  RPS2-R  RIN4 KO-RP  RIN4 KO-LP  LBb1.3  RIN4-F  RIN4-R  GABI-8474  GABI-DN35  GABI-07dk  GABI-DN39  GABI-10dk  Exo70B1-2 F  Exo70B1-2 R  **RT-PCR primers**  CACC-RIN4-F  RIN4-RT-R  Exo70B1-RT-F4  Exo70B1-RT-R4  Exo70B2-RT-F4  Exo70B2-RT-R4  IPP2-RT-F  IPP2-RT-R | ATGGATCCACAACAACAACAACTC  AGAAGCCGTCCGATGAGCTTTCCC  GTTCTGAACTTGTACTACAGTTAC  CGGATATTCCGCAGACAATCTTGG  GGTACCCAAATATCTCTGATTCATATCAAATCAGTTAC  ATATGAGAAGCCGAGAAGAGAGCGAGTTGA  ATTTTGCCGATTTCGGAAC  CACCATGGCACGTTCGAATGTACCAAA  TCATTTTCCTCCAAAGCCAAAGC  ATAATAACGCTGCGGACATCTACATTTT  ATACATGTTACAAATCCTCCCTGC  AGTCTGTGTTCTCGGATCAGTTTT  ATCCCTCTAATCGCTTCTCCTAAC  CTTACAGATTCTCTCCGGTTATGG  CCTTCATTTTCTTCCCGTGGTAGT  TTCGTTTATGGAGGTTTGTCGTGG  CACCATGGCACGTTCGAATGTACCAAA  GGTTCTGGAAGAAGGAGGATGAG  GGGAGATACATTGTTCAGAAAG  CCCAGCATCTTATTCCATGAGC  GGCGATCTAAACGCGATCGCTG  CTTCCTCCATACTTAGTCCTCTG  TGCTCTTCATCGTGCGAGACGT  GCTCCTTCAGCTCTTCCCGGC | |

**
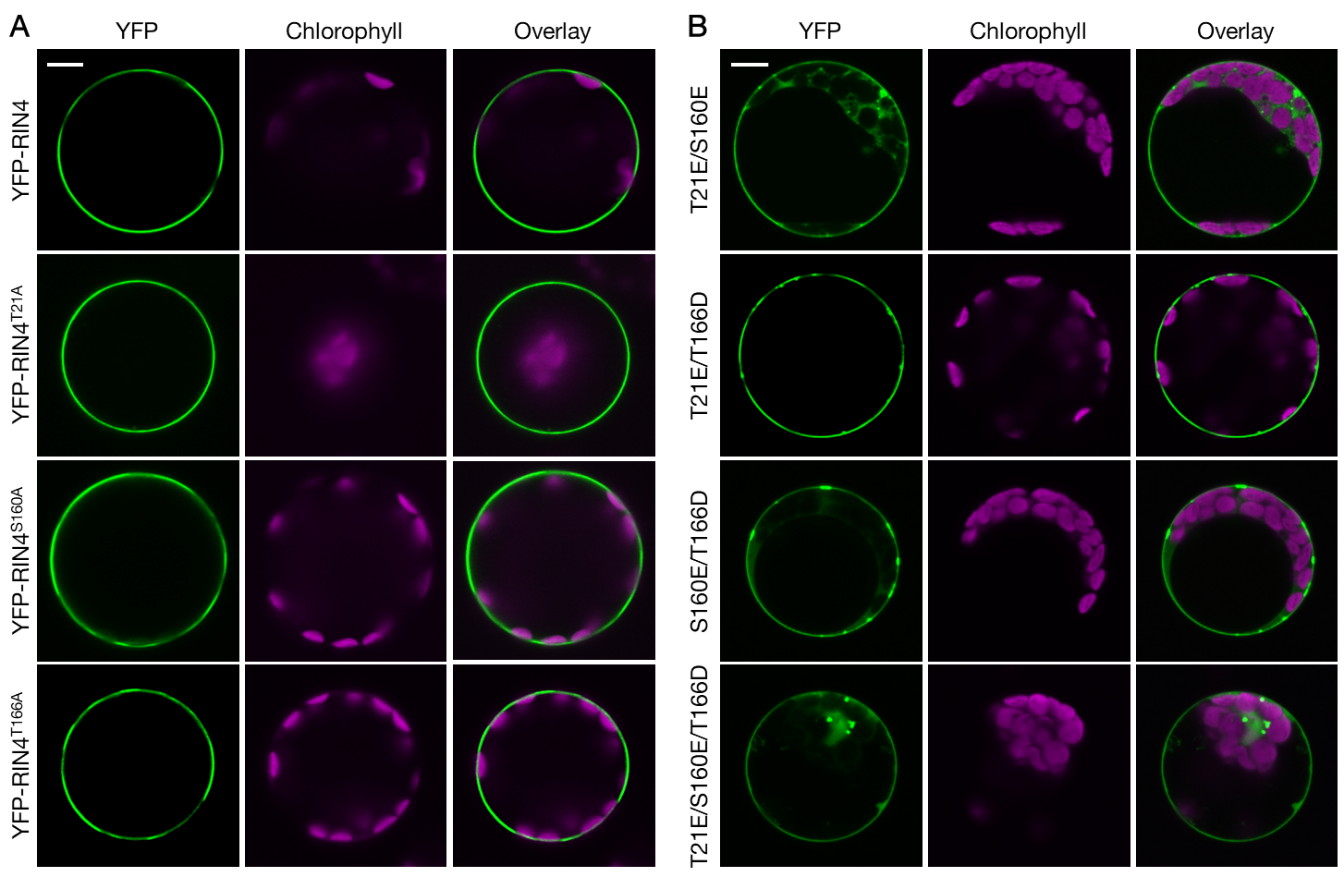
**

**FIGURE S1 The subcellular localization of phospho-dead and hyperphosphorylation mutants of RIN4.**

(A) All tested phospho-dead mutants of RIN4 show PM-localization in protoplast extracted from WT Col-0. Transient expression of YFP-labelled RIN4 phospho-dead mutants (RIN4^T21A^, RIN4^S160A^, RIN4^S166A^). Protoplasts were imaged using a Nikon C2 CLSM (40X oil-immersion objective, images at zoom 3). YFP fluorescence is pseudo-colored green, and chlorophyll is magenta. Images were captured for at least three biological replicates and there were at least 15 protoplasts of each line checked. Bar = 10 μm.

(B) Hyperphosphorylation of RIN4 results in disruption of PM-localization. Transient expression of YFP fused RIN4 phosphomimetic variants in protoplast extracted from WT Col-0. Protoplasts have been imaged by a Nikon C2 CLSM (40X oil-immersion objective, images at zoom 3). YFP fluorescence in green and chlorophyll in magenta. Images were captured for at least three biological replicates and there were at least 15 protoplasts of each line checked. T21E/S160E, YFP-RIN4^T21E/S160E^; T21E/T166D, YFP-RIN4^T21E/T166D^; S160E/T166D, YFP-RIN4^S160E/T166D^; T21E/S160E/T166D, YFP-RIN4^T21E/S160E/T166D^. Bars = 10 μm.


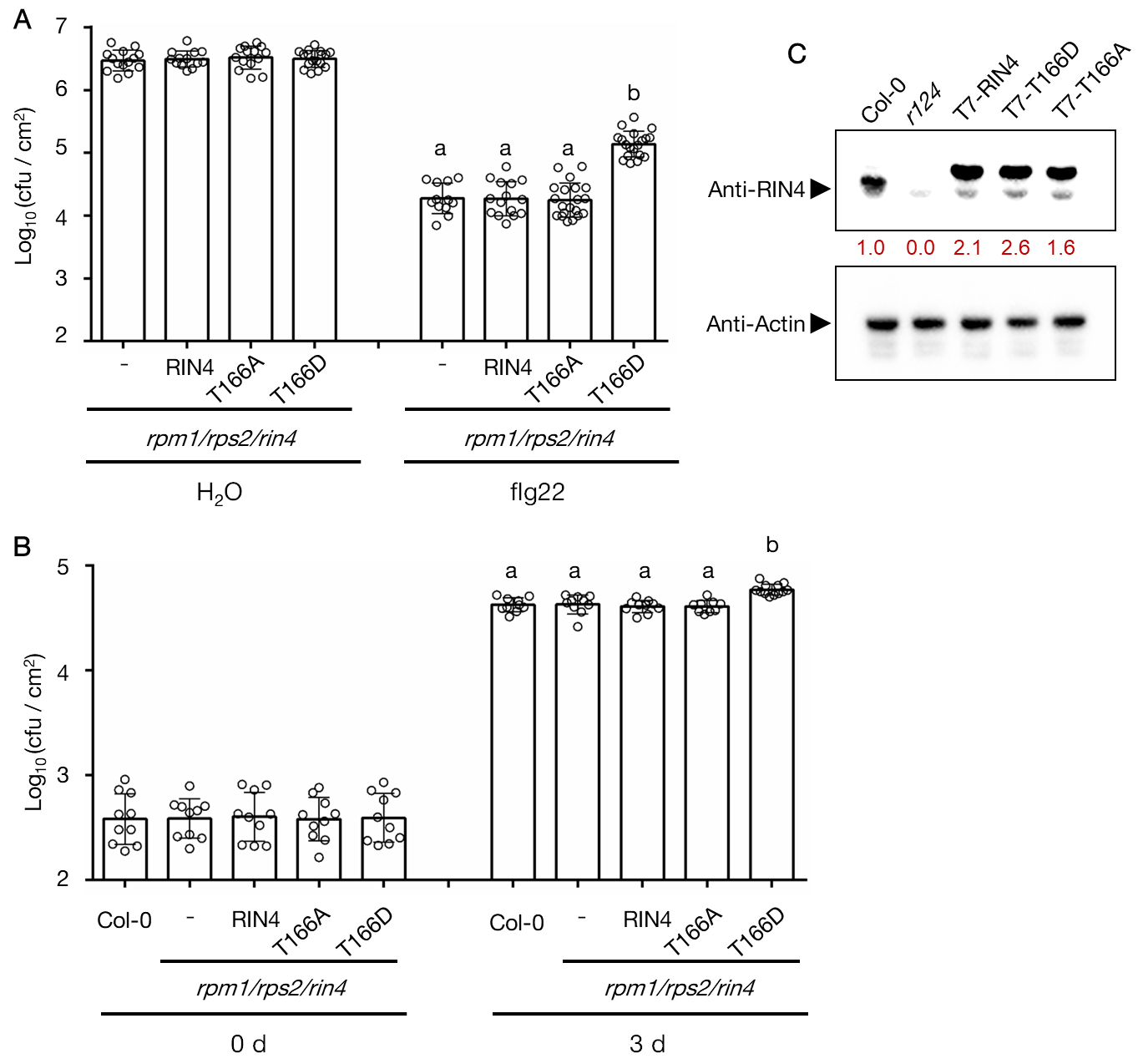


**FIGURE S2 The phosphorylation status of RIN4 at Thr-166 represses PTI.**

(A) The effect of flg22-priming assay on PTI was repressed in the RIN4^T166D^ transgenic line. *Pst* DC3000 (EV) bacteria were hand infiltrated at 1 x 10^6^ cfu/mL 24 hr post 1 μM flg22-treatment. Leaves were harvested 2 days later (n ≥ 12/genotype and treatment). Significance was determined by one-way ANOVA with Tukey-Kramer HSD with 95% confidence (RIN4, T166A to T166D: P < 0.0001; *rpm1/rps2/rin4* to T166D: P = 0.0001). Error bars represent SD. RIN4, *RIN4 native promoter::T7-RIN4* in the *rpm1/rps2/rin4* genetic background; T166A, *RIN4 native promoter::T7-RIN4^T166A^* in the *rpm1/rps2/rin4* genetic background; T166D, *RIN4* native promoter::T7-RIN4^T166D^ in the *rpm1/rps2/rin4* genetic background. All experiments were repeated three times with similar results.

(B) RIN4^T166D^ has a negative role in plant immunity against *Pst* DC3000 *hrcC*^-^. Leaves were infiltrated with *Pst* DC3000 *hrcC^-^* at 1 x 10^6^ cfu/mL to induce PTI, and samples were taken at 0-day and 3 days post infiltration (n ≥ 10/genotype and treatment). Significance was determined by one-way ANOVA with Tukey-Kramer HSD with 95% confidence (Col-0, *rpm1/rps2/rin4,* RIN4, T166A to T166D: P < 0.0001). Error bars represent SD. All experiments were repeated three times with similar results.

(C) No difference of RIN4 protein level in the total protein extracts of transgenic lines expressing RIN4 isoforms in the *rpm1/rps2/rin4* genetic background. Immunoblotting analysis was performed on total protein extracts of WT Col-0, *r124*, and transgenic lines expressing *RIN4* native promoter driven T7 tagged RIN4 isoforms (RIN4, RIN4^T166D^ or RIN4^T166A^) in *rpm1/rps2/rin4* with an anti-RIN4 antibody (rabbit anti-RIN4 antibody, homemade, 1:2000). Plant actin was used as protein marker. The relative quantities of the protein levels of RIN4 variants were calculated using Fiji and are indicated in red. *r124* = *rpm1/rps2/rin4* triple mutant. All experiments were repeated three times with similar results.

**
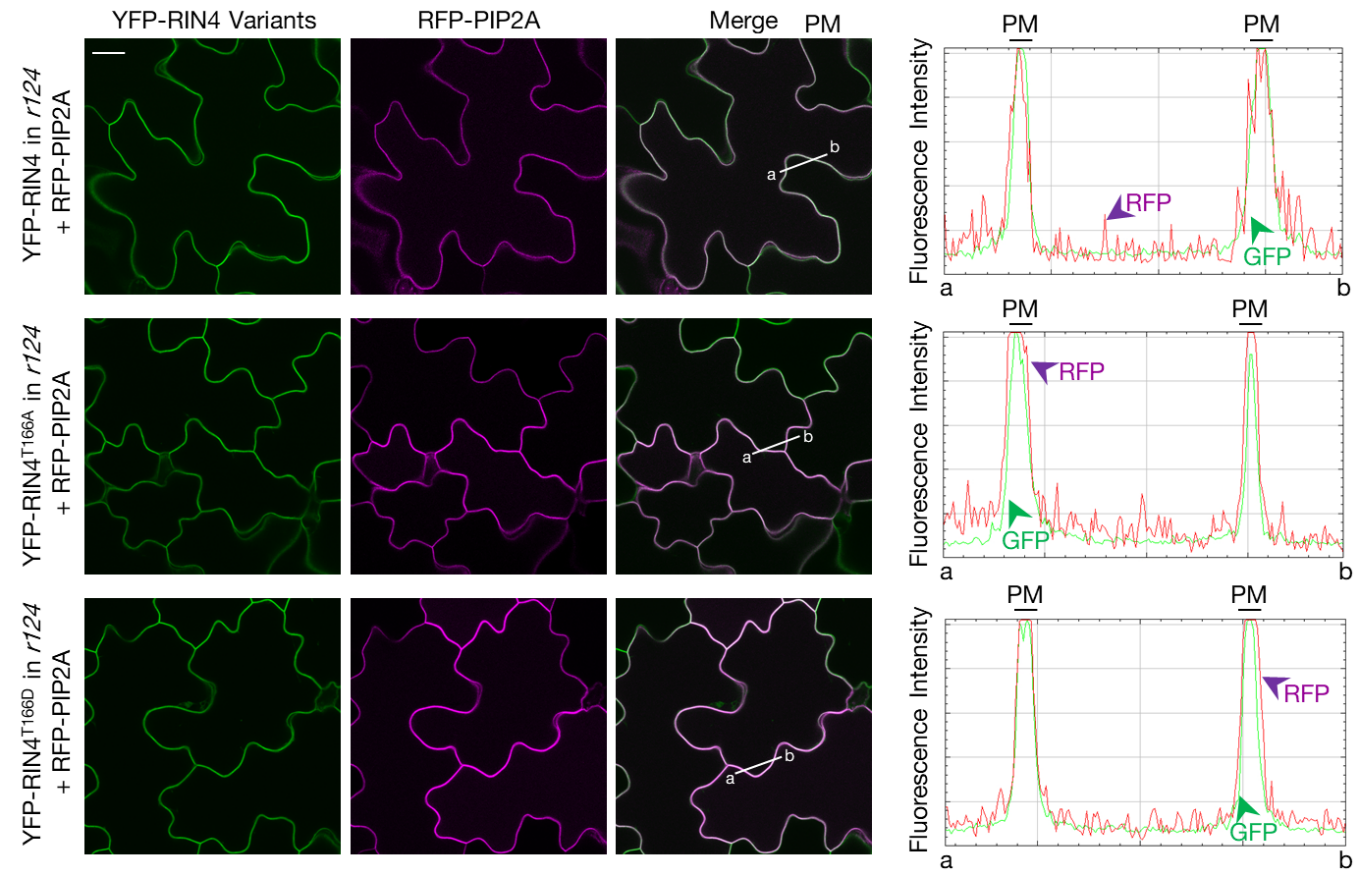
**

**FIGURE S3 YFP-RIN4 co-localizes with the plasma membrane marker.**

Fluorescence images showing the subcellular localization of YFP fused RIN4 variants and RFP fused plasma membrane marker PIP2A. The panels from left to right display fluorescence images that showing YFP labelled RIN4 variants (RIN4, RIN4^T166A^, RIN4^T166D^), RFP labelled PIP2A, overlay fluorescence of YFP and RFP, and fluorescence intensity graphs which show YFP (green) and RFP (magenta) signals measured along the white line from a to b separately. The images represent a single focal plane (40X oil-immersion objective, Nikon A1R-TIRF-STORM). The 14-d-old plants were used. Images were captured for at least three biological replicates and there were at least 5 plants of each line checked. PM, plasma membrane. *r124* = *rpm1/rps2/rin4* triple mutant. Bar = 20 μm.

**
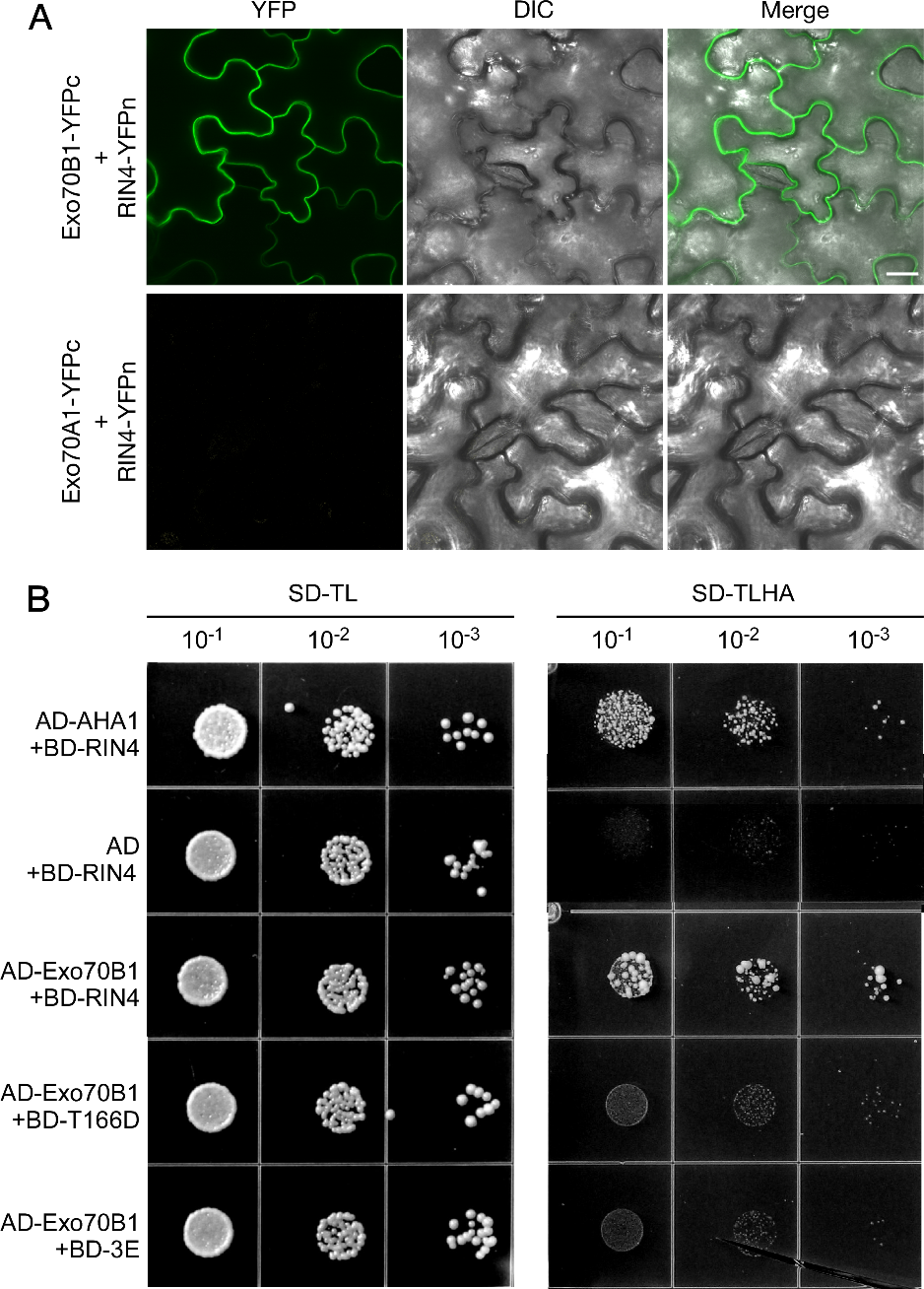
**

**FIGURE S4 The interaction between RIN4 and Exo70B1 is abolished by the phospho-mimetic mutations on RIN4.**

(A) Confirmation of the interaction between RIN4 and Exo70B1 using Split-YFP analysis. Bimolecular fluorescence complementation (BiFC) of RIN4 and Exo70B1 in *N. benthamiana* leaves transiently expressed from co-infiltrated agrobacterium-transformed constructs. Co-infiltration of RIN4-YFPn and Exo70A1-YFPc were used as negative control. Images were captured for three biological replicates. Bar = 20 μm.

(B) RIN4 T166 phosphorylation mutant abolishes the interaction between RIN4 and Exo70B1 in yeast-two hybrid assay. Empty vectors pDEST22 were used as negative controls, the interaction between pDEST22-AHA1 and pDEST32-RIN4 was used as positive control. Experiment was performed for three biological replicates. T166D stands for RIN4^T166D^, 3E = RIN4^T21E/S160E/T166D^.


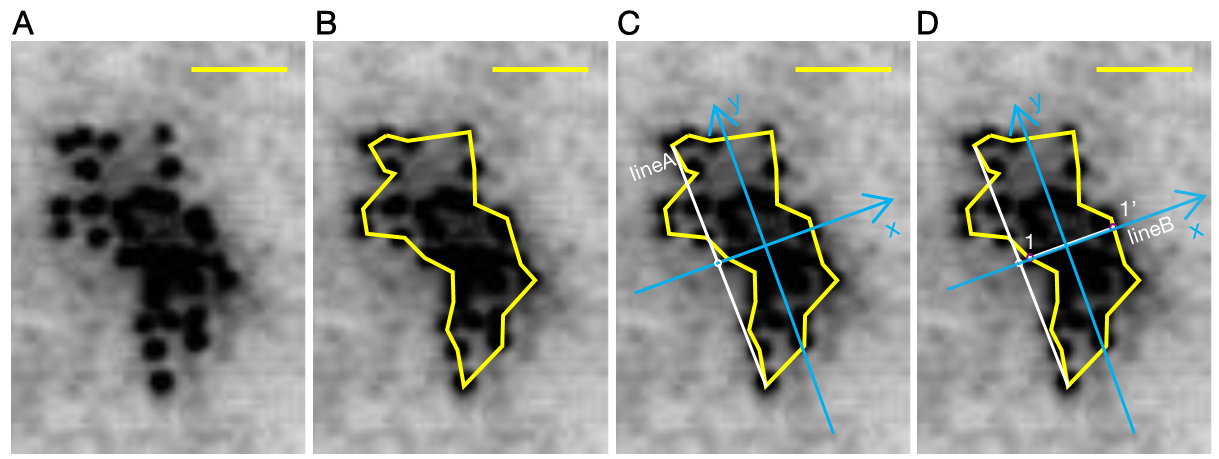


**FIGURE S5 Schematic diagram of evaluation of aggregation size.**

An aggregation particle (A) was selected from immunogold labeling image and a closed graph was made by linking the outmost nanoparticles with lines (B). Drawing a line between two furthest points of this graph, naming it as lineA, measuring the length of lineA, and putting a rectangular coordinate system on the graph, whose y-axis parallels lineA and x-axis passes through the midpoint of lineA (C). Labelling the points where x- axis crossed graph as 1 and 1’ (D) and measuring the distance between 1-1’ (lineB). The average length of line A and lineB was used as a standard to evaluate the size of the aggregation. Bars = 200 nm.

**
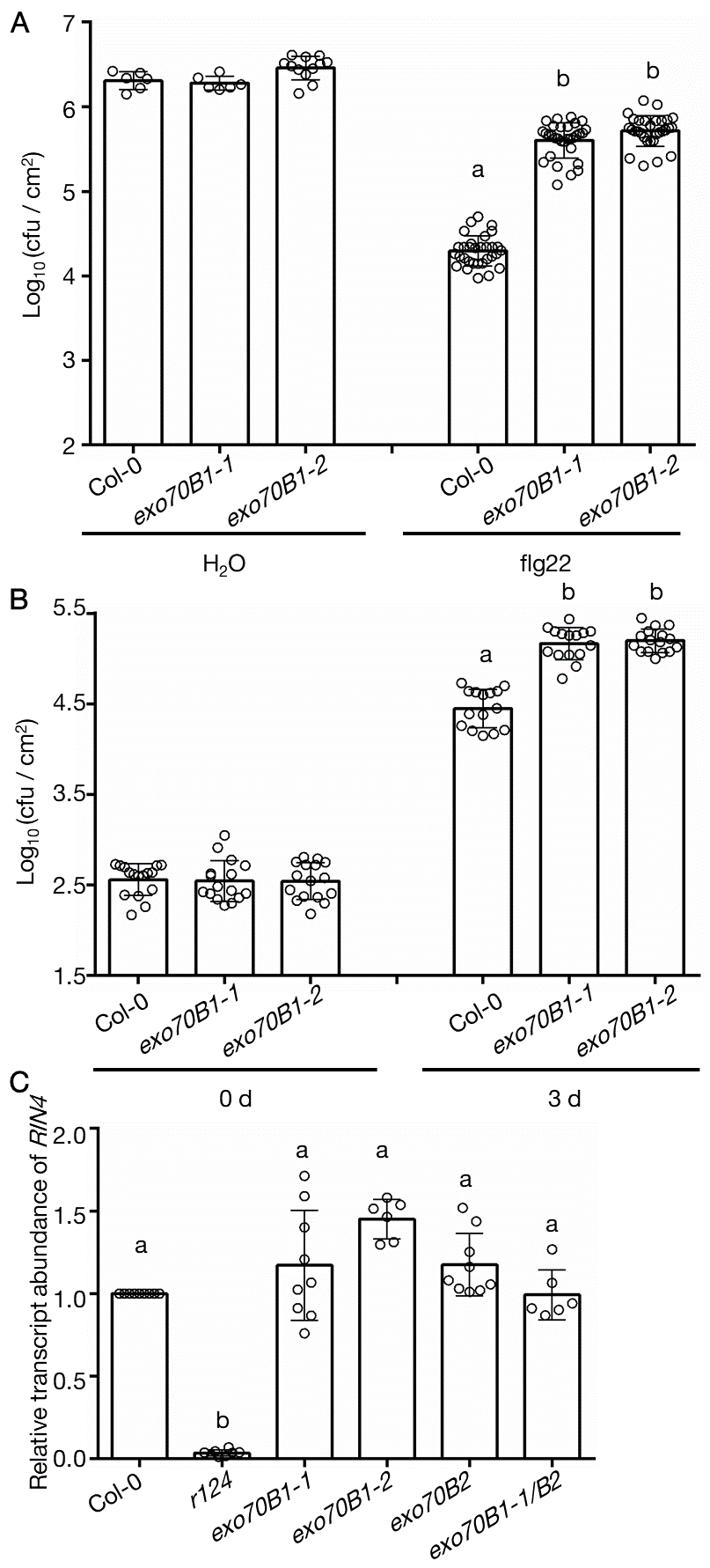
**

**FIGURE S6 Two alleles of *exo70B1* show similar repressed PTI responses.**

(A) *exo70B1-1* and *exo70B1-*2 show reduced PTI phenotypes following flg22-priming (n ≥ 15/genotype and treatment). Significance was determined by one-way ANOVA with Tukey-Kramer HSD with 95% confidence (Col-0 to *exo70B1-1*: P < 0.0001; to *exo70B1-*2: P < 0.0001). Error bars represent SD. All experiments were repeated three times with similar results.

(B) Leaves were infiltrated with *Pst* DC3000 *hrcC^-^* at 1 x 10^6^ cfu/mL to induce PTI, and samples were taken at 0-day post infiltration and 3 days post infiltration (n ≥ 6/genotype and treatment). Significance was determined by one-way ANOVA with Tukey-Kramer HSD with 95% confidence (Col-0 to *exo70B1-1*: P < 0.0001; to *exo70B1-*2: P < 0.0001). Error bars represent SD. All experiments were repeated three times with similar results.

(C) Transcription level of *RIN4* in WT Col-0, *r124*, and *exo70* mutants (n ≥ 6/genotype). Significance was determined by one-way ANOVA with Tukey-Kramer HSD with 95% confidence (Col-0 to *exo70B1-2*: P < 0.0007; to *r124*: P < 0.0001). Error bars represent SD. All experiments were repeated three times with similar results. Relative expression values were determined using *IPP2* gene (At3g02780) as a reference and the comparative Ct method (2^-ΔΔ^*^C^*^t^). Primers used are listed in Supplementary Table 1.
